# Force-dependent focal adhesion assembly and disassembly: A computational study

**DOI:** 10.1371/journal.pcbi.1011500

**Published:** 2023-10-06

**Authors:** Kailas Shankar Honasoge, Zeynep Karagöz, Benjamin T. Goult, Haguy Wolfenson, Vanessa L. S. LaPointe, Aurélie Carlier

**Affiliations:** 1 Department of Cell Biology–Inspired Tissue Engineering, MERLN Institute for Technology-Inspired Regenerative Medicine, Maastricht University, Maastricht, the Netherlands; 2 School of Biosciences, University of Kent, Canterbury, United Kingdom; 3 Department of Genetics and Developmental Biology, Rappaport Faculty of Medicine, Technion – Israel Institute of Technology, Haifa, Israel; Clemson University, UNITED STATES

## Abstract

Cells interact with the extracellular matrix (ECM) via cell–ECM adhesions. These physical interactions are transduced into biochemical signals inside the cell which influence cell behaviour. Although cell–ECM interactions have been studied extensively, it is not completely understood how immature (nascent) adhesions develop into mature (focal) adhesions and how mechanical forces influence this process. Given the small size, dynamic nature and short lifetimes of nascent adhesions, studying them using conventional microscopic and experimental techniques is challenging. Computational modelling provides a valuable resource for simulating and exploring various “what if?” scenarios *in silico* and identifying key molecular components and mechanisms for further investigation. Here, we present a simplified mechano-chemical model based on ordinary differential equations with three major proteins involved in adhesions: integrins, talin and vinculin. Additionally, we incorporate a hypothetical signal molecule that influences adhesion (dis)assembly rates. We find that assembly and disassembly rates need to vary dynamically to limit maturation of nascent adhesions. The model predicts biphasic variation of actin retrograde velocity and maturation fraction with substrate stiffness, with maturation fractions between 18–35%, optimal stiffness of ∼1 pN/nm, and a mechanosensitive range of 1-100 pN/nm, all corresponding to key experimental findings. Sensitivity analyses show robustness of outcomes to small changes in parameter values, allowing model tuning to reflect specific cell types and signaling cascades. The model proposes that signal-dependent disassembly rate variations play an underappreciated role in maturation fraction regulation, which should be investigated further. We also provide predictions on the changes in traction force generation under increased/decreased vinculin concentrations, complementing previous vinculin overexpression/knockout experiments in different cell types. In summary, this work proposes a model framework to robustly simulate the mechanochemical processes underlying adhesion maturation and maintenance, thereby enhancing our fundamental knowledge of cell–ECM interactions.

## Author summary

The immediate environment of a cell is vastly different in healthy and diseased tissue and influences numerous intracellular processes. Mechanical and chemical properties of the extracellular matrix (ECM) impact cellular processes through various pathways, enabling cells to ‘sense’ their environment and respond accordingly. Cell-ECM interactions occur through adhesions and are mediated by transmembrane proteins called integrins, which, depending on the ECM properties, can recruit, directly or indirectly, >200 proteins and transduce external signals to intracellular chemical signals. However, how adhesions assemble, grow, and disassemble is poorly understood. Here, using a differential equation-based mechano-chemical computational model, we investigate how substrate stiffness influences adhesion formation, maturation and disassembly. We explicitly include the role of talin and vinculin-based reinforcement in these processes. The model we present is robust and can be fine-tuned for particular cell-types and signalling mechanisms. The simulations predict the highest amount of maturation on substrates of an intermediate, ‘optimum’ stiffness, and that (dis)assembly rates need to change dynamically for cells to establish this ‘optimum’. In summary, our results highlight the importance of vinculin availability in adhesion maturation and reinforcement and aid our understanding of cell adhesion formation and mechanotransduction.

## Introduction

**Fig 1:**
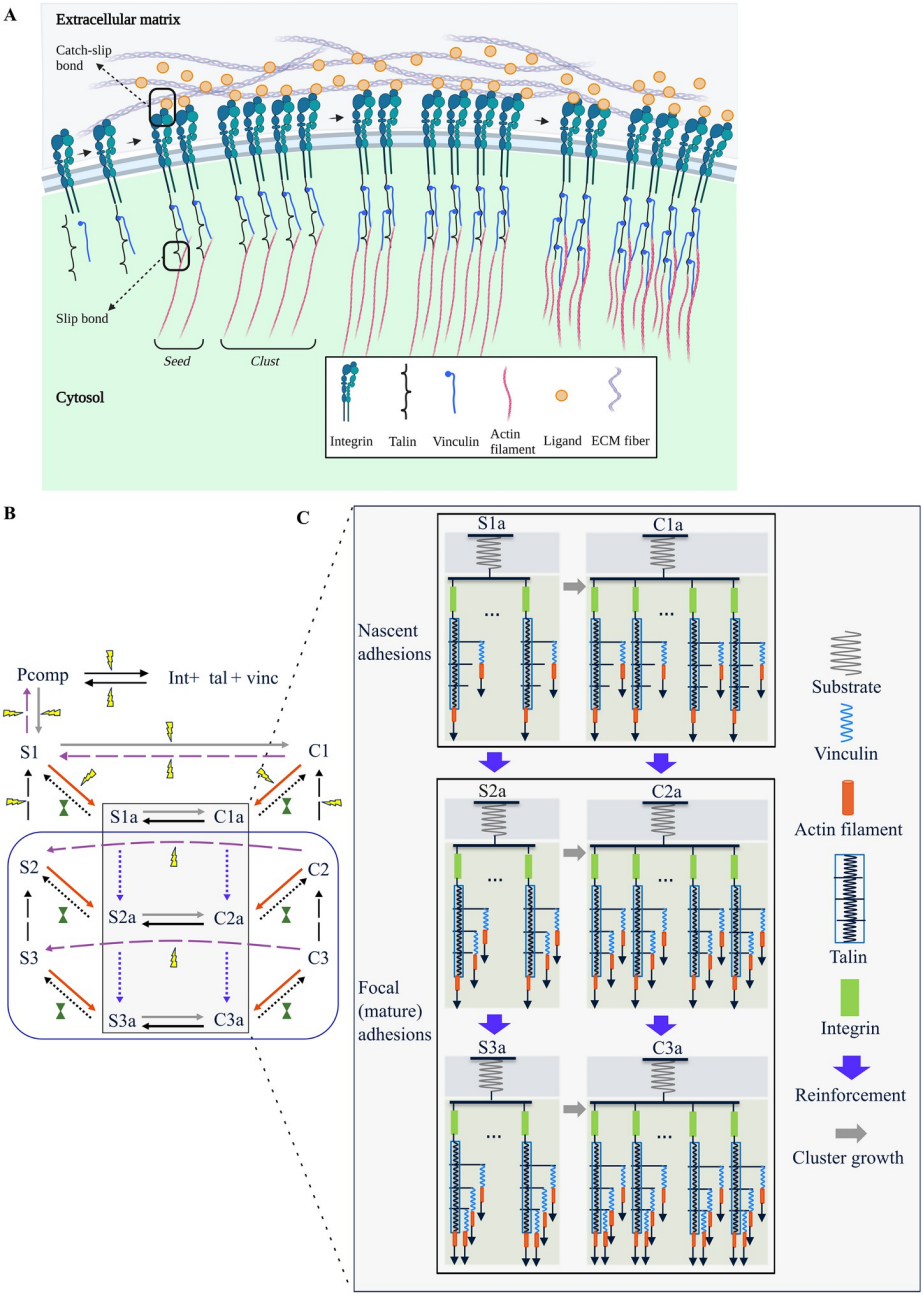
Overview of the processes modelled in this study. (A)—Integrins bind to talin and vinculin in a precomplexation step, then form a small cluster, termed ‘seed’. Seeds can dimerise to form larger clusters, termed ‘clusts’. Actin filaments pull on talin and vinculin causing cryptic vinculin-binding sites on talin to be exposed, promoting more vinculin recruitment. This chain can then break at the integrin–ligand catch-slip bond or the talin–actin slip bond (black boxes). See Table 1 for a detailed description of the terminology. (B)—Overview of reactions in the model. Int, tal, and vinc refer to concentrations of integrins, talin and vinculin respectively. Black rectangle encloses the reinforcement reactions (expanded further in Fig 1C). Grey arrows represent clust formation reactions. Red arrows represent actin binding reactions. Dotted arrows represent force-dependent reactions—blue dotted: reinforcement, black dotted: actin unbinding. Dashed arrows represent adhesion disassembly reactions, black dashed: talin refolding, purple dashed: cluster breakdown. Yellow lightning bolts indicate rates that undergo signal-dependent rate modification (SDRM), dark green solid hourglasses represent rates that undergo time-dependent rate modification (TDRM). The rate constants undergoing signal-dependent modifications are driven to zero after ∼158 s leaving active only the lower part of the model, enclosed in the blue box, representing adhesions that will undergo further maturation. (C)—Talin and vinculin are modelled as Hookean springs (also see Fig A in S1 Appendix). In this model, to capture the process of reinforcement, a maximum of three vinculin binding events occur sequentially (blue arrows) at different points along the talin rod, thereby increasing the stiffness of individual integrin–talin–vinculin spring systems. Clustering is modelled as an increase in the number of integrin–talin–vinculin spring systems in parallel (grey arrows). This figure was created using BioRender.com.

Direct contact between cells and the extracellular matrix (ECM) through adhesions is a crucial component of multicellular organisms [1]. Integrins are transmembrane ECM receptor proteins that assemble as non-covalently bonded heterodimers with *α* and *β* subunits [2]. The integrin ectodomain binds ECM ligands while the cytoplasmic tail is indirectly linked to the actomyosin cytoskeleton of the cell forming a supramolecular assembly or ‘clutch’ [3–6]. This indirect link consists of a dynamic network of over 200 proteins, collectively termed the ‘integrin adhesome’ [7, 8]. Central to integrin function are the dynamics and balance of extra- and intracellular forces [9] which drive the force-dependent evolution of the integrin adhesion complexes (IACs) [7] leading to changes in their size and composition. *In vitro* studies have shown that adhesion assembly is a multi-step process where integrins are first activated by binding to intracellular adaptor protein molecules such as talin [10–12] and/or to an ECM ligand [13]. Once activated, integrins cluster at the site of adhesion, independent of force and substrate rigidity, to form nascent adhesions (NAs) [14–16]. Then, NAs either undergo disassembly or force-dependent maturation by the recruitment of other adaptor proteins such as vinculin, to form focal adhesions (FA) (Fig 1A) [17, 18]. These three major steps of adhesion assembly also overlap in time and are not strictly sequential. Understanding interactions between key proteins of the integrin adhesome and force generation will provide valuable insight into cell-ECM interactions, with consequences for developmental biology. A better understanding of cellular responses and signalling can potentially highlight new therapeutic targets, and improve engineered substrates to better mimic biological tissue, thus advancing regenerative medicine.

In addition to questions pertaining to adhesion (dis)assembly, adhesion maturation is also a complex process influenced by the mechanical properties of the substrate [19, 20], force-dependent conformational changes [21–25], different catch and slip bond strengths [5, 26, 27] and intracellular forces [20, 28, 29]. How changes in these mechanical factors affect the biochemical composition of adhesions, and which factors determine the decision to mature a particular NA remains unclear.

Given the constraints and challenges of experimental studies, computational modelling can be a valuable resource. Many computational models of cell–ECM interactions have been developed since the first molecular-clutch model by Chan and Odde (2008) [4] that explained filopodial traction dynamics on compliant substrates [30–34]. Elosegui-Artola and colleagues have extended the Chan and Odde model to include adhesion reinforcement through increases in integrin density [35] and multiple integrin types [36]. Integrin-based Rho signalling [34] and reversible cross-links in the actin filament network [37] have also been included in previous studies by other groups. More recently, Venturini and Saez (2023) [38] have developed an extensive multi-scale model of molecular clutch-driven adhesion mechanics. All these models explore adhesion formation, growth, and the influence of substrate stiffness and actomyosin forces on traction forces, but they are discrete models that simulate a relatively small number of individual particles. They also do not account for the increase in clutch stiffness after the recruitment of vinculin and do not consider the disassembly processes to be dynamic and active. In addition, these models give little information about the changes to the overall biochemical composition of adhesions in the cell during the process of maturation of NAs to FAs.

In this study, we developed a new model using ordinary differential equations (ODEs) to describe the biochemical composition of cell–ECM adhesions over time based on mechanical properties like substrate stiffness, adaptor protein stiffness, actomyosin-generated forces, and bond characteristics. Using our model, we studied the fraction of NAs that have the potential to become mature FAs under different mechanical circumstances. Overall, the results from this study shed light on the mechanotransduction mechanisms underlying adhesion maturation and disassembly. This model also provides a reliable starting point to model the larger focal adhesome with over 200 identified proteins [7].

## Methods

### Differential equation model

We developed an ODE-based model that captures changes in the biochemical composition of cell–ECM adhesions based on the mechanical properties of the environment and intracellular proteins. Below we shortly describe the particular phases of the adhesion maturation process—formation of integrin-talin-vinculin precomplexes, formation and growth of precomplex clusters, actin binding and unbinding, adhesion reinforcement with vinculin, and adhesion breakdown—and how they are modelled (Fig 1B provides an overview of all the species in the system and their interactions). For each subprocess (in bold), we give a brief explanation and also label the corresponding terms in the differential equations (Eqs 1–17). Unless otherwise mentioned, reactions are reversible with forward rate constants having ‘f’ in the subscript and reverse rate constants having ‘r’. Reactions follow mass-action kinetics unless mentioned otherwise. When referring to concentrations in the text, they are written between square brackets (e.g., [S3a]), and when referred to as a species they are written as is (e.g., S3a). A detailed explanation of the reactions and parameters can be found in Text A and Table A in S1 Appendix. Table 1 provides an overview of the terminology used throughout the manuscript.

**Table 1:** Terminology used in this manuscript.

Term	Description
Integrin-Adaptor Protein Complex (IAPC)	Assembly of integrin, talin and vinculin. Three possible IAPCs with talin bound to one, two, or three vinculin molecules are considered.
Pcomp	A pre-complex—an individual IAPC with talin bound to a single vinculin molecule.
Seed—(*Sx*)	A cluster of 25 IAPCs bound parallelly (Fig 1C). Denoted by *Sx*, where *x* is the number of vinculin molecules bound to talin in each IAPC that makes up the seed, (*x* ∈ {1, 2, 3}).
Clust—(*Cx*)	A cluster of 50 IAPCs bound parallelly (cluster size taken from [16]) (Fig 1C). Denoted by *Cx*, where *x* ∈ {1, 2, 3}) is the number of vinculin molecules bound to talin in each IAPC that makes up the clust.
Order—(*x* ∈ {1, 2, 3})	The order of a seed/clust is indicative of the number of vinculin molecules bound to talin in each IAPC that makes up the seed/clust. Low, mid and high order seeds/clusts refer to seeds/clusts made of IAPCs containing one, two or three vinculin molecules respectively.
Clutch	An umbrella term that refers to a supramolecular assembly of integrins and adaptor proteins that can function as a molecular clutch between the substrate and the cell (Fig 1C). In this model, both seeds and clusts are capable of this function.
Actin Bound (AB)—(*Sxa*, *Cxa*)	Talin and vinculin have actin-binding sites allowing clutches to bind to actin, experience a force and, consequently, an extension. Seeds and clusts that are bound to actin are said to be actin bound (AB). Denoted by *Sxa* and *Cxa* respectively (*x* ∈ {1, 2, 3}).
Actin Unbound (AUB)	The talin–actin and vinculin–actin bonds break at certain force thresholds resulting in the clutches being unbound from actin. These are actin unbound (AUB) clutches. Denoted by *Sx* and *Cx* (*x* ∈ {1, 2, 3}). AUB clutches do not experience any force or extension.

Adhesion assembly starts with integrin activation. In this study, we model *α*_5_*β*_1_ integrins and assume they are activated. We also assume that the ligand spacing on the substrate is sufficiently close for integrin clusters to form.

#### Pcomp formation and dissociation

Activated integrins, [Int], bind to talin, [tal], and vinculin, [vinc], forming pre-complexes [Pcomp], a necessary step for adhesion maturation [18] (Text A in S1 Appendix).

#### Seed formation and dimerisation

Up to 50 IAPCs cluster independent of substrate rigidity and tension to form NAs [16]. Here, as a simplification, the growth of clusters to the maximal size (50 IAPCs) occurs in two stages—first, a small cluster of 25 IAPCs, termed ‘seed’ (denoted by ‘Sx’ (Fig 1C, Table 1) is formed, and a second stage where seeds dimerise, forming a large cluster with 50 IAPCs, termed ‘clust’ (denoted by ‘Cx’ (Fig 1C, Table 1, Text A in S1 Appendix)). Here, *x* ∈ {1,2,3} denotes the number of vinculin molecules in the individual IAPCs.

When a clutch is AUB, the stretched talin is likely to refold [24, 39]. We assume this makes it very unlikely for AUB seeds of mid- and high- order (S2, S3) to dimerise. AB seeds of all orders (S1a, S2a, S3a) can dimerise to form AB clusts (C1a, C2a, and C3a) (grey arrows in Fig 1B).

#### Actin binding/unbinding

Actin-unbound (AUB) seeds and clusts can bind to actin through the actin-binding sites on talin and vinculin, giving AB seeds and clusts (denoted by ‘Sxa’ and ‘Cxa’ respectively, (Text A in S1 Appendix)) that can stretch to different extents (based on the value of *x*) and hence transmit varying magnitudes of force (Fig 1C).

While the baseline actin-binding rate is *k_act_* for all actin-binding reactions, signalling molecules such as focal adhesion kinase (FAK), Src and ERK play a role in adhesion turnover, and their inhibition leads to more maturation [40]. To implement a similar mechanism to stop indefinite adhesion formation and maturation, we introduce in the model a signal-dependent rate modification (SDRM) (see ‘Signal dependent rate modification (SDRM)’ for details).

Depending on the force on the integrin-ligand (catch-slip) bonds [41] and the talin-actin (slip) bonds [5], the force-chain between the cell and the substrate can break at either of these bonds. We capture these phenomena through force-dependent (and consequently substrate rigidity-dependent) actin unbinding rates (black dotted arrows in Fig 1B, also see Text A in S1 Appendix for details). These bonds may also rupture due to random thermodynamic fluctuations before the clutches can reach their maximum force-carrying capacity, reducing the total force exerted by the clutches. To account for the spontaneous clutch unbinding in a continuous framework, we introduce a time-dependent rate modification (TDRM) (see ‘Force-dependent actin-unbinding and time-dependent rate modification (TDRM)’ for details).

#### Reinforcement

The AB clutches form a mechanical link between the substrate and the actin cytoskeleton, enabling force transmission and protein unfolding. Up to eleven cryptic vinculin-binding sites (VBS) are uncovered when talin is stretched and unfolded [42, 43], leading to reinforcement by vinculin recruitment. The rate of talin unfolding and reinforcement occurring depends on the force experienced by the AB clutch, and increases with increasing force, similar to the Bell model [24, 44, 45] (for more details see section ‘Adhesion reinforcement rates’). In this simplified model, two vinculin-reinforcement events are considered, one from low- to mid-order clutches (S1a to S2a and C1a to C2a), followed by one from mid- to high-order clutches (S2a to S3a and C2a to C3a) (see Text A in S1 Appendix for a detailed explanation). Thus, in this model, the talin rod can be bound to at least 1 and at most 3 vinculin molecules. With this framework, we consider low-order clutches (S1, S1a, C1, C1a (Fig 1C)) to represent NAs, and mid- and high-order clutches (S2, S2a, C2, C2a, S3, S3a, C3, C3a, (Fig 1C)) to represent more mature stages of adhesions, indicative of the fraction of NAs that mature into FAs (see Section ‘NA formation is rigidity- and force-independent’ for reasoning). Reinforcement is modelled as a single-step reaction where simultaneous recruitment of 25 (for seeds) and 50 (for clusts) vinculin molecules respectively occurs (indicated by blue dotted arrows in Fig 1B). The order of these reactions with respect to vinculin, however, was chosen to be 2 (Eqs 3, 8, 9 and 10), to account for the effects of possible intermediate stages in the reactions.

#### Refolding and breakdown

In the absence of sufficient force, adhesions disassemble because of mechanical and chemical signals [46]. Here, we model two parallel processes of disassembly, namely 1) talin refolding (black dashed arrows in Fig 1B) leading to the loss of vinculin from, and weakening of, clutches, and 2) breakdown of AUB clusters into seeds and Pcomp (purple dashed arrows in Fig 1B) leading to reduced force carrying capacity of the adhesions (Text A in S1 Appendix). These are irreversible reactions.

To account for the mechanical aspects of adhesion assembly, the substrate–integrin–adaptor protein system was formulated as a system of Hookean springs. When clutches bind to the actin filaments, they provide resistance to the motion of actin filaments until bond rupture, caused either randomly or because the catch/slip bond force threshold is reached. We assume that the force exerted by myosin II motors on actin filaments is balanced by the drag force arising due to the viscosity of the cytoplasm. Thus, in the absence of integrin-mediated forces on actin filaments, they move with a constant retrograde velocity (see Text A in S1 Appendix). As a continuous ODE framework is used, we consider the same actin retrograde velocity for all clutches. The force on a clutch depends on its stiffness and extension (according to Hooke’s law). The stiffness of a clutch depends on the number of constituent IAPCs and the number of vinculin molecules in each IAPC (equivalent spring constants are calculated, see Text A in S1 Appendix). The total force exerted on the actin filament network thus depends on the number of AB clutches of each type and their stiffnesses. Since we use a continuum approach to account for the abundance of each species, we discretise concentrations of AB clutches by assuming a volume of 1 *μm*^3^ to calculate the total force (see Text A in S1 Appendix).

Together, the above-described processes result in the following set of differential equations:

$$\begin{array}{c} \frac{d[int]}{dt}=-\underset{Pcomp\;\;formation\;\;and\;\;dissociation}{\underset{︸}{k_{1f}\cdot \left[int\right]\cdot \left[tal\right]\cdot \left[vinc\right]+k_{1r}\cdot \left[Pcomp\right]}} \end{array}$$ (1)

$$\begin{array}{c} \frac{d[tal]}{dt}=-\underset{Pcomp\;\;formation\;\;and\;\;dissociation}{\underset{︸}{k_{1f}\cdot \left[int\right]\cdot \left[tal\right]\cdot \left[vinc\right]+k_{1r}\cdot \left[Pcomp\right]}} \end{array}$$ (2)

$$\begin{array}{cc} \frac{d[vinc]}{dt} & =-\underset{Pcomp\;\;formation\;\;and\;\;dissociation}{\underset{︸}{k_{1f}\cdot \left[int\right]\cdot \left[tal\right]\cdot \left[vinc\right]+k_{1r}\cdot \left[Pcomp\right]}}\\ & +25\cdot (-\underset{S1a\;\;\underset{↔}{reinforcement}\;\;S2a}{\underset{︸}{k_{7f}\cdot \left[S1a\right]\cdot {\left[vinc\right]}^{2}+k_{7r}\cdot \left[S2a\right]}}-\underset{S2a\;\;\underset{↔}{reinforcement}\;\;S3a}{\underset{︸}{k_{8f}\cdot \left[S2a\right]\cdot {\left[vinc\right]}^{2}+k_{8r}\cdot \left[S3a\right]}}\\ & +\underset{S3\overset{\text{refolding}}{\to }\;\;S2}{\underset{︸}{k_{17f}\cdot \left[S3\right]}}+\underset{S2\overset{\text{refolding}}{\to }\;\;S1}{\underset{︸}{k_{18f}\cdot \left[S2\right]}})\\ & +50\cdot (-\underset{C1a\;\;\underset{↔}{reinforcement}\;\;C2a}{\underset{︸}{k_{12f}\cdot \left[C1a\right]\cdot {\left[vinc\right]}^{2}+k_{12r}\cdot \left[C2a\right]}}-\underset{C2a\;\;\underset{↔}{reinforcement}\;\;C3a}{\underset{︸}{k_{13f}\cdot \left[C2a\right]\cdot vinc^{2}+k_{13r}\cdot \left[C3a\right]}}\\ & +\underset{C3\overset{\text{refolding}}{\to }\;\;C2}{\underset{︸}{k_{19f}\cdot \left[C3\right]}}+\underset{C2\overset{\text{refolding}}{\to }\;\;C1}{\underset{︸}{k_{20f}\cdot \left[C2\right]}}) \end{array}$$ (3)

$$\begin{array}{cc} \frac{d[Pcomp]}{dt} & =\underset{Pcomp\;\;formation\;\;and\;\;dissociation}{\underset{︸}{k_{1f}\cdot \left[int\right]\cdot \left[tal\right]\cdot \left[vinc\right]-k_{1r}\cdot \left[Pcomp\right]}}\\ & -25\cdot \underset{Seed\;\;formation}{\underset{︸}{\left(k_{2f}\cdot {\left[Pcomp\right]}^{2}-k_{2r}\cdot \left[S1\right]\right)}} \end{array}$$ (4)

$$\begin{array}{cc} \frac{d[S1]}{dt} & =\underset{Seed\;\;formation}{\underset{︸}{k_{2f}\cdot {\left[Pcomp\right]}^{2}-k_{2r}\cdot \left[S1\right]}}-\underset{S1\;\;\underset{↔}{dimerisation}\;\;C1}{\underset{︸}{2\cdot (k_{3f}\cdot {\left[S1\right]}^{2}-k_{3r}\cdot \left[C1\right])}}\\ & -\underset{Actin\;\;binding/unbinding}{\underset{︸}{k_{4f}\cdot \left[S1\right]+k_{4r}\cdot \left[S1a\right]}}+\underset{S2\overset{\text{refolding}}{\to }\;\;S1}{\underset{︸}{k_{18f}\cdot \left[S2\right]}} \end{array}$$ (5)

$$\begin{array}{c} \frac{d[S2]}{dt}=-\underset{Actin\;\;binding/unbinding}{\underset{︸}{k_{5f}\cdot \left[S2\right]+k_{5r}\cdot \left[S2a\right]}}+\underset{S3\overset{\text{refolding}}{\to }\;\;S2}{\underset{︸}{k_{17f}\cdot \left[S3\right]}}-\underset{S2\overset{\text{refolding}}{\to }\;\;S1}{\underset{︸}{k_{18f}\cdot \left[S2\right]}}+\underset{C2\overset{\text{breakdown}}{\to }\;\;S2}{\underset{︸}{2\cdot k_{22f}\cdot \left[C2\right]}} \end{array}$$ (6)

$$\begin{array}{c} \frac{d[S3]}{dt}=-\underset{Actin\;\;binding/unbinding}{\underset{︸}{k_{6f}\cdot \left[S3\right]+k_{6r}\cdot \left[S3a\right]}}-\underset{S3\overset{\text{refolding}}{\to }\;\;S2}{\underset{︸}{k_{17f}\cdot \left[S3\right]}}+\underset{C3\overset{\text{breakdown}}{\to }\;\;S3}{\underset{︸}{2\cdot k_{21f}\cdot \left[C2\right]}} \end{array}$$ (7)

$$\begin{array}{cc} \frac{d[S1a]}{dt} & =\underset{Actin\;\;binding/unbinding}{\underset{︸}{k_{4f}\cdot \left[S1\right]-k_{4r}\cdot \left[S1a\right]}}-\underset{S1a\;\;\underset{↔}{reinforcement}\;\;S2a}{\underset{︸}{k_{7f}\cdot \left[S1a\right]\cdot {\left[vinc\right]}^{2}+k_{7r}\cdot \left[S2a\right])}}\\ & -2\cdot (\underset{S1a\;\;\underset{↔}{dimerisation}\;\;C1a}{\underset{︸}{k_{14f}\cdot {\left[S1a\right]}^{2}-k_{14r}\cdot \left[C1a\right]}}) \end{array}$$ (8)

$$\begin{array}{cc} \frac{d[S2a]}{dt} & =\underset{Actin\;\;binding/unbinding}{\underset{︸}{k_{5f}\cdot \left[S2\right]-k_{5r}\cdot \left[S2a\right]}}+\underset{S1a\;\;\underset{↔}{reinforcement}\;\;S2a}{\underset{︸}{k_{7f}\cdot \left[S1a\right]\cdot {\left[vinc\right]}^{2}-k_{7r}\cdot \left[S2a\right]}}\\ & -\underset{S2a\;\;\underset{↔}{reinforcement}\;\;S3a}{\underset{︸}{k_{8f}\cdot \left[S2a\right]\cdot {\left[vinc\right]}^{2}+k_{8r}\cdot \left[S3a\right]}}-2\cdot \left(\underset{S2a\;\;\underset{↔}{dimerisation}\;\;C2a}{\underset{︸}{k_{15f}\cdot {\left[S2a\right]}^{2}-k_{15r}\cdot \left[C2a\right]}}\right) \end{array}$$ (9)

$$\begin{array}{cc} \frac{d[S3a]}{dt} & =\underset{Actin\;\;binding/unbinding}{\underset{︸}{k_{6f}\cdot \left[S3\right]-k_{6r}\cdot \left[S3a\right]}}+\underset{S2a\;\;\underset{↔}{reinforcement}\;\;S3a}{\underset{︸}{k_{8f}\cdot \left[S2a\right]\cdot {\left[vinc\right]}^{2}-k_{8r}\cdot \left[S3a\right]}}\\ & -2\cdot (\underset{S3a\;\;\underset{↔}{dimerisation}\;\;C3a}{\underset{︸}{k_{16f}\cdot {\left[S3a\right]}^{2}-k_{16r}\cdot \left[C3a\right]}}) \end{array}$$ (10)

$$\begin{array}{c} \frac{d[C1]}{dt}=\underset{S1\;\;\underset{↔}{dimerisation}\;\;C1}{\underset{︸}{2\cdot k_{3f}\cdot {\left[S1\right]}^{2}-k_{3r}\cdot \left[C1\right]}}-\underset{Actin\;\;binding/unbinding}{\underset{︸}{k_{9f}\cdot \left[C1\right]+k_{9r}\cdot \left[C1a\right]}}+\underset{C2\overset{refolding}{\to }C1}{\underset{︸}{k_{20f}\cdot \left[C2\right]}} \end{array}$$ (11)

$$\begin{array}{c} \frac{d[C2]}{dt}=-\underset{Actin\;\;binding/unbinding}{\underset{︸}{k_{10f}\cdot \left[C2\right]+k_{10r}\cdot \left[C2a\right]}}+\underset{C3\overset{\text{refolding}}{\to }\;\;C2}{\underset{︸}{k_{19f}\cdot \left[C3\right]}}-\underset{C2\overset{\text{refolding}}{\to }\;\;C1}{\underset{︸}{k_{20f}\cdot \left[C2\right]}}-\underset{C2\overset{\text{breakdown}}{\to }\;\;S2}{\underset{︸}{k_{22f}\cdot \left[C2\right]}} \end{array}$$ (12)

$$\begin{array}{c} \frac{d[C3]}{dt}=-\underset{Actin\;\;binding/unbinding}{\underset{︸}{k_{11f}\cdot \left[C3\right]+k_{11r}\cdot \left[C3a\right]}}-\underset{C3\overset{\text{refolding}}{\to }\;\;C2}{\underset{︸}{k_{19f}\cdot \left[C3\right]}}-\underset{C3\overset{\text{breakdown}}{\to }\;\;S3}{\underset{︸}{k_{21f}\cdot \left[C3\right]}} \end{array}$$ (13)

$$\begin{array}{cc} \frac{d[C1a]}{dt} & =\underset{Actin\;\;binding/unbinding}{\underset{︸}{k_{9f}\cdot \left[C1\right]-k_{9r}\cdot \left[C1a\right]}}-\underset{C1a\;\;\underset{↔}{reinforcement}\;\;C2a}{\underset{︸}{k_{12f}\cdot \left[C1a\right]\cdot {\left[vinc\right]}^{2}+k_{12r}\cdot \left[C2a\right]}}\\ & +\underset{S1a\;\;\underset{↔}{dimerisation}\;\;C1a}{\underset{︸}{k_{14f}\cdot {\left[S1a\right]}^{2}-k_{14r}\cdot \left[C1a\right]}} \end{array}$$ (14)

$$\begin{array}{cc} \frac{d[C2a]}{dt} & =\underset{Actin\;\;binding/unbinding}{\underset{︸}{k_{10f}\cdot \left[C2\right]-k_{10r}\cdot \left[C2a\right]}}+\underset{C1a\;\;\underset{↔}{reinforcement}\;\;C2a}{\underset{︸}{k_{12f}\cdot \left[C1a\right]\cdot {\left[vinc\right]}^{2}-k_{12r}\cdot \left[C2a\right]}}\\ & -\underset{C2a\;\;\underset{↔}{reinforcement}\;\;C3a}{\underset{︸}{k_{13f}\cdot \left[C2a\right]\cdot {\left[vinc\right]}^{2}+k_{13r}\cdot \left[C3a\right]}}+\underset{S2a\;\;\underset{↔}{dimerisation}\;\;C2a}{\underset{︸}{k_{15f}\cdot {\left[S2a\right]}^{2}-k_{15r}\cdot \left[C2a\right]}} \end{array}$$ (15)

$$\begin{array}{cc} \frac{d[C3a]}{dt} & =\underset{Actin\;\;binding/unbinding}{\underset{︸}{k_{11f}\cdot \left[C3\right]-k_{11r}\cdot \left[C3a\right]}}+\underset{C2a\;\;\underset{↔}{reinforcement}\;\;C3a}{\underset{︸}{k_{13f}\cdot \left[C2a\right]\cdot {\left[vinc\right]}^{2}-k_{13r}\cdot \left[C3a\right]}}\\ & +\underset{S3a\;\;\underset{↔}{dimerisation}\;\;C3a}{\underset{︸}{k_{16f}\cdot {\left[S3a\right]}^{2}-k_{16r}\cdot \left[C3a\right]}} \end{array}$$ (16)

Baseline parameter values and rate constants can be found in Table A in S1 Appendix. We refer the reader to Text A in S1 Appendix for detailed descriptions of all reactions in the model and the underlying reasoning. Below, we highlight the novel methodological approaches (signal- and time-dependent rate modification (SDRM and TDRM)), and provide brief explanations of a few mathematical formulations and assumptions that are used in this model.

### Signal dependent rate modification (SDRM)

Nascent adhesions (NAs) form in large numbers and most are disassembled within a time scale of a few minutes [17, 47]. As the cell protrudes, the distance between the cell membrane and the NAs increases, and actin depolymerization rates are higher away from the cell membrane [48]. Thus, numerous NAs may be supported near the cell membrane but in the absence of this scaffold, many NAs disassemble. Various signal cascades also regulate adhesion disassembly. Signalling molecules such as focal adhesion kinase (FAK), Src and ERK kinases play a role in adhesion turnover, and their inhibition leads to more maturation [40]. However, most studies have investigated the effects of signalling molecules on the turnover of FAs and not NAs [49, 50], and the exact mechanical or chemical triggers for NA disassembly remain elusive [51]. NA assembly at the cell front, maturation, and disassembly away from the leading edge occur constantly due to above-mentioned mechanisms. In this study, we focus on one cycle of NA formation and investigated the differences in NA maturation on different substrate stiffnesses. To implement a mechanism to stop indefinite adhesion formation and maturation, it is hypothesized that there exists a signal molecule of which a minimum concentration, *signal_thresh_*, is required for new NA formation and low-order AUB clutches (S1, C1) to bind actin for maturation. The concentration [signal] of this molecule is initially high and decreases at an arbitrary rate following Michaelis-Menten kinetics given by:

$$\begin{array}{c} \frac{d[signal]}{dt}=\frac{-k_{23_{vmax}}\cdot \left[signal\right]}{k_{23_{KM}}+\left[signal\right]} \end{array}$$ (17)

Initial estimates for values of the maximum velocity $k_{23_{vmax}}$ and the Michaelis constant $k_{23_{KM}}$ were based on those reported in the literature for FAK Tyr-397 phosphorylation in the presence of ATP [52] but were adjusted such that the concentration of [signal] reaches the *signal_thresh_* in 58 s, which is the approximate duration of the NA assembly phase as measured in experiments [4]. When the [signal] falls below *signal_thresh_*, it is analogous to a signalling pathway being activated, and particular reaction rate constants (*k*_1*f*_, *k*_1*r*_, *k*_2*f*_, *k*_2*r*_, *k*_3*f*_, *k*_3*r*_, *k*_4*f*_, *k*_9*f*_, *k*_18*f*_, *k*_20*f*_, *k*_21*f*_, *k*_22*f*_, indicated by yellow lighting bolts in Fig 1B) are modified as detailed in Text A in S1 Appendix. Thus, the rate of decay of [signal] determines the amount of time available before NA disassembly starts in which adhesion maturation can occur. Note that the model behaviour does not change if we assume the opposite i.e., [signal] increases over time and there is an upper limit for its concentration beyond which actin binding does not occur (Fig C.B in S1 Appendix).

### Force-dependent actin-unbinding and time-dependent rate modification (TDRM)

A cell–ECM force chain is broken if either the integrin–ligand (catch-slip) bond [41] or the talin–actin (slip) bond [5] ruptures as a result of reaching the respective force thresholds or due to random thermodynamic fluctuations. This is described in detail in Text A in S1 Appendix. To capture the combined dynamics of the catch-slip and slip bonds, as well as the effect of random bond ruptures, the actin unbinding rates (dark green hourglasses in Fig 1B) are defined as follows:

$$\begin{array}{c} \left\{k_{4\text{r}},k_{5\text{r}},k_{6\text{r}},k_{9\text{r}},k_{10\text{r}},k_{11\text{r}}\right\}=A\cdot e^{-b\cdot F_{clutch}}+C\cdot e^{d\cdot F_{clutch}}+k_{TDRM}\cdot k_{slip_{UL}}\cdot e^{\frac{F_{clutch}}{F_{th_{i}}}} \end{array}$$ (18)

where *F_clutch_* is the force on an individual IAPC in the clutch (see Text A in S1 Appendix where *F_clutch_* is described in detail). The first and second terms, where *A* and *C* are scaling factors and *b* and *d* control the force-dependency, describe the integrin–ligand catch-slip bond, and the third term describes the talin-actin slip bond where $F_{{th}_{i}}$ is bond rupture threshold for a given clutch type (*i* ∈ {1, 2, 3}, refer Table A in S1 Appendix) and $k_{{slip}_{UL}}$ is the unloaded dissociation rate. *k_TDRM_* is the time-dependent rate modification (TDRM) factor that is required to qualitatively account for the reduction in the total force caused by spontaneous clutch unbinding events. *k_TDRM_* is given by:

$$\begin{array}{c} k_{TDRM}=1+k_{sens}\cdot t_{clutch}\cdot dt \end{array}$$ (19)

where *t_clutch_* is the number of simulated time-steps that a clutch remains actin-bound, *dt* is the time-step and *k_sens_* is a parameter that determines the magnitude of the influence. In short, this definition captures the decreasing likelihood of an AB clutch remaining AB for long periods of time. It has been shown that integrin-ligand bonds undergo cyclic mechanical reinforcement (CMR) leading to longer lifetimes [53]. This implies that on soft substrates where the force-loading rate is low, integrin-ligand bonds experience fewer force cycles in a given time period compared to stiff substrates and consequently are more likely to break on soft substrates. Previous studies model CMR with an increased bond-dissociation rate at low forces [35, 38]. Here, TDRM can capture the effects of these phenomena. TDRM affects softer substrates, where clutches take longer to reach their force thresholds, more than stiff substrates.

When an AB clutch experiences a force equal to its force threshold, it unbinds from actin and becomes an AUB clutch. Thus, the concentration of the AB clutch is set to 0, and the concentration of its AUB counterpart is increased by the same amount.

### Adhesion reinforcement rates

The rate at which the talin rod unfolds increases with applied force and has been described in previous studies by the Bell model [24, 44, 45]. Here, the Bell formulation was adapted such that the rate increases exponentially with force until the vinculin binding force threshold *F_vb_* is reached, beyond which it remains constant. The rate is given by:

$$\begin{array}{c} k_{unfold}=\{\begin{array}{cc} k_{unfold_{UL}}\cdot e^{k_{UF}\cdot \frac{F_{clutch}}{F_{vb_{i}}}} & \text{if}\;F_{clutch}\leq F_{vb_{i}}\\ k_{unfold_{UL}}\cdot e^{k_{UF}} & \text{if}\;F_{clutch}>F_{vb_{i}} \end{array} \end{array}$$ (20)

where $k_{{unfold}_{UL}}$ is the rate of unloaded talin unfolding, *k_UF_* is a parameter controlling force-dependence, *F_clutch_* is the force experienced by an individual IAPC in the clutch, and $F_{{vb}_{i}}$ is the vinculin binding force threshold, with *i* ∈ {1, 2} corresponding to the first and second vinculin binding steps. Here, $F_{{vb}_{1}}=5\;\text{pN}$ and $F_{{vb}_{2}}=12\;\text{pN}$ [24, 42]. Vinculin binding is assumed to occur instantaneously once the VBS is uncovered [45, 54], and hence the rates of reinforcement were determined based on the force-dependent unfolding kinetics of talin as observed in single-molecule experiments using magnetic tweezers [42]. A detailed description of the method used for curve-fitting can be found in Text A in S1 Appendix.

### Range of substrate rigidities

The stiffness of the ECM, generally measured in terms of Young’s modulus, varies across three orders of magnitude, between 1–3 kPa in the brain, 23–42 kPa in muscular tissue, 1000 kPa-860 MPa in blood vessels, to 15–40 GPa in bone [55]. In this study, however, we consider spring constants, which can be converted to Young’s moduli based on a few assumptions as detailed in [34, 36] and summarized in Text A in S1 Appendix. Accordingly, we restrict the scope of the main investigations and predictions to a stiffness range equivalent to 0.1–130 kPa and only qualitatively discuss the results up to 1000 kPa. This is in line with previous computational and experimental studies which have also primarily studied adhesion mechanobiology in similar ranges of substrate rigidities [4, 36].

### Local sensitivity analysis

As the model included many parameters whose values were either estimated or adapted to fit experimental data, a local sensitivity analysis was performed. The range of values tested for each parameter was the baseline value (Table A in S1 Appendix) ±10% and ±20%. To quantify the influence, two different metrics were used as outcomes, namely 1) maturation fraction: the concentration of integrins in mid- and high-order AB and AUB clutches ([S2]+[S2a]+[S3]+[S3a]+[C2]+[C2a]+[C3]+[C3a]) at equilibrium (the last time point), and 2) the optimal stiffness: the substrate stiffness with the lowest mean actin retrograde velocity. Outcome 1 represents the total fraction of integrins in the system that made it beyond the initial force-independent stage of adhesion formation, indicative of the fraction of NAs that mature into FAs. Outcome 2 represents an overall influence on the system as it quantifies the mean force exerted during the length of the simulation for a range of substrate stiffnesses. In addition, cells are known to be able to tune their mechanosensitive ranges to adapt to their environments, an aspect on which outcome 2 can shed light. As different parameters may have different levels of influence based on the substrate stiffness, the sensitivity of outcome 1 to each parameter was evaluated for four substrate stiffnesses (*k_sub_* = 0.1, 1, 10, 100 pN/nm).

Parameter sensitivity analysis was performed on 21 parameters (Text B, Fig I and Fig J in S1 Appendix), and the ones with the highest influence or of key importance are presented in the main text. The parameter sensitivity for a parameter *p* for an outcome *i* was calculated as follows:

$$\begin{array}{c} \begin{array}{c} Sensitivity_{p,i}=\frac{|Outcome_{i}\left(p+\Delta p\right)-Outcome_{i}\left(p\right)|}{Outcome_{i}\left(p\right)}/\frac{\Delta p}{p} \end{array} \end{array}$$ (21)

where *Outcome*_*i*_(*p* + Δ*p*) represents the value of the outcome metric with the changed parameter value, *Outcome_i_(p)* is the value of the outcome metric with the baseline parameter value, and Δ*p* and *p* are the change in the parameter and the baseline parameter value respectively.

### Initial conditions

The initial concentrations of integrins, talin and vinculin were assumed to be equal and set to 1 *μ*M for simplicity, and that of all other species were set to 0. Vinculin was assumed to be abundantly available within the cytoplasm and thus modelled at a constant concentration of 1 *μ*M throughout the simulation.

### Simulation steps

**Fig 2:**
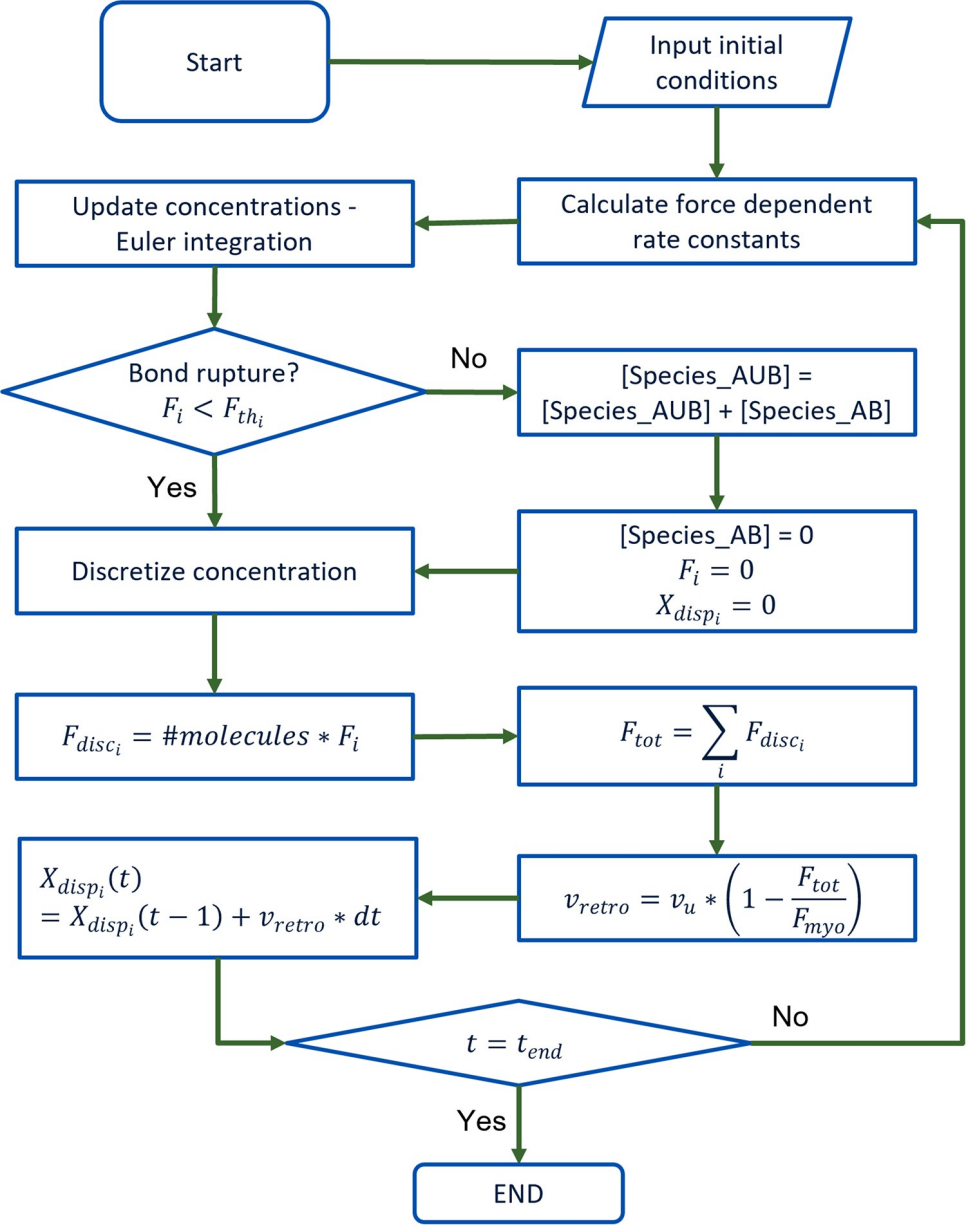
Schematic showing the flowchart for simulation and force quantification.

All simulations were run for 600 s. Euler’s forward integration method was used to solve the ODEs with a time step *dt* of 5 ms as used in previous computational studies [4, 35]. For mass conservation steps, see Text A in S1 Appendix. The steps of integration and the order of updates (Fig 2) of the different aspects of the model are as follows:

1. Force-dependent rate constants are calculated. In particular, the following rates are evaluated at the current force:
  - First reinforcement rates: (*k*_7*f*_, *k*_12*f*_) using Eq 20.
  - Second reinforcement rates: (*k*_8*f*_, *k*_13*f*_) using Eq 20.
  - Signal-dependent rate modification: (*k*_1*f*_, *k*_1*r*_, *k*_2*f*_, *k*_2*r*_, *k*_3*f*_, *k*_3*r*_, *k*_4*f*_, *k*_9*f*_, *k*_18*f*_, *k*_20*f*_, *k*_21*f*_, *k*_22*f*_) are updated as detailed in Text A in S1 Appendix.
  - Catch-slip bond rates with time-dependent rate modification: (*k*_4*r*_, *k*_5*r*_, *k*_6*r*_, *k*_9*r*_, *k*_10*r*_, *k*_11*r*_) using Eqs 18 and 19.
2. Concentrations are updated based on current rate constants by solving the differential equations listed above.
3. The slip bond threshold is checked for each clutch type
  - If the slip bond threshold is reached, the force on the clutch is reset to 0.
  - The concentration of the actin-bound form of the clutch is converted to the actin-unbound form.
4. The total force exerted by actin-bound clutches is calculated based on discretised concentrations using eq. S53.
5. Retrograde velocity, *v_retro_*, is updated based on the current total force in the system using eq. S49.
6. All substrate-clutch spring systems are extended by an amount *v*_*retro*_ ⋅ *dt*.
7. Force on each clutch is updated using eq. S51.

## Results

To explore the influence of mechanical properties like substrate stiffness, adaptor protein stiffness, actomyosin-generated forces and bond characteristics on adhesion maturation, we developed a computational model that captures the overall changes to the IAC compositions as adhesions form and mature. The model, based on ODEs, consists of a single compartment that represents a patch of the cell where adhesions form, and considers three core components: integrins, talin and vinculin, from which 14 other species are made. The dynamic NA/FA maturation is modelled by a total of 22 reactions (explained in Methods and Text A, Tables A and B in S1 Appendix) that largely represent three distinct processes (Fig 1): (i) adhesion formation, (ii) reinforcement and growth, and (iii) adhesion disassembly.

Using our mechanochemical computational model, we find that dynamic rates of assembly and disassembly, which are likely regulated by biochemical signalling events, are essential to determine the subset of NAs that mature. The model was found to satisfy mass conservation (Text B, Fig B in S1 Appendix).

### NA formation is rigidity- and force-independent

**Fig 3:**
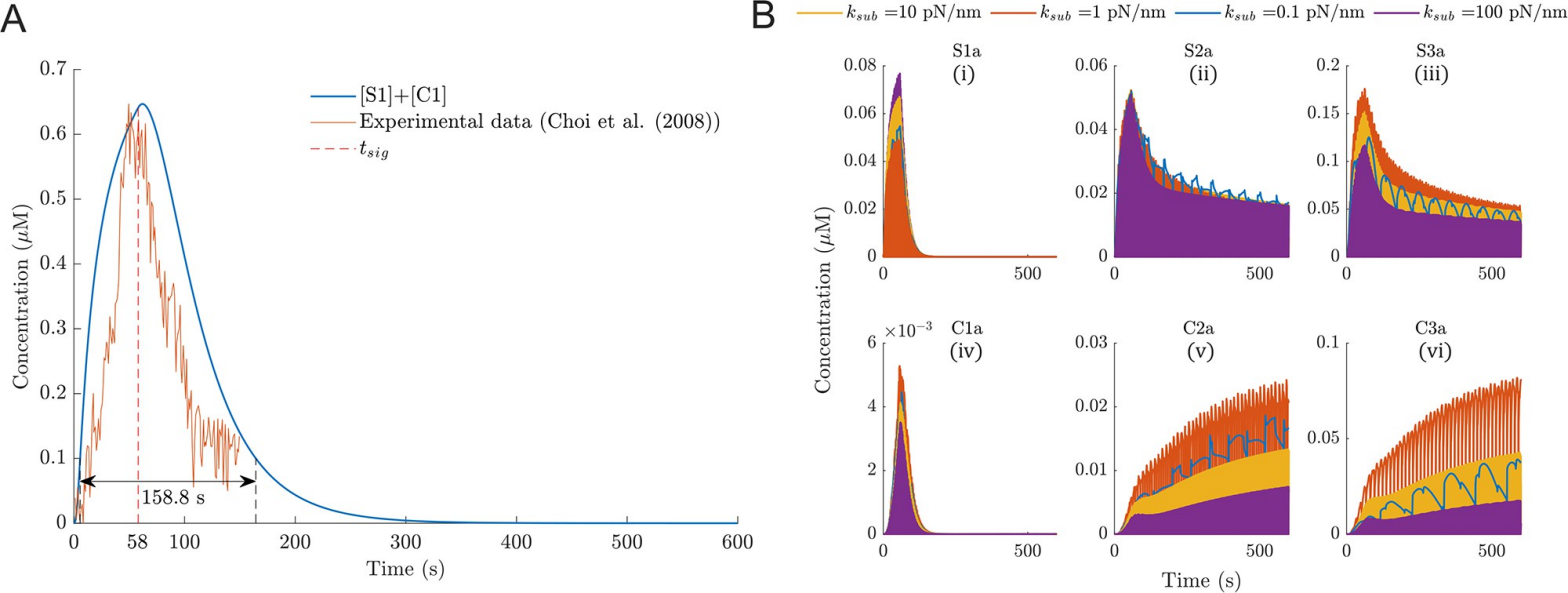
While NA formation is substrate stiffness independent, maturation is influenced considerably by stiffness. (A)—Concentration over time of species in the model that represent NAs (S1 and C1). The curves for all tested substrate rigidities overlap and hence appear as a single (blue) line. The vertical dotted line marks the time point when the signal threshold is crossed and hence new NA formation reduces. (B)—Concentrations over time of all the actin-bound species. Species representing NAs (S1a, C1a) increase initially before being driven to 0 after the signal concentration drops below the threshold. The highest levels of maturation occur on substrate of moderate stiffness (*k_sub_* = 1 pN/nm).

When only pre-complex, initial seed and clust formation reactions (Rx1, Rx2 and Rx3) were active (see Text B, Table B in S1 Appendix) the concentration of seeds and clusts for all substrate stiffnesses tested were equal, in line with previous experimental evidence showing that NA formation is substrate rigidity-independent [16] (Fig 3A). This is the result of the rigidity- and force-independent rate constants (*k*_1*f*_, *k*_1*r*_, *k*_2*f*_, *k*_2*r*_, *k*_3*f*_, *k*_3*r*_) for reactions Rx1, Rx2 and Rx3. Thus, the concentration of seeds and clusts formed only depends on the initial concentrations of [int], [tal], and [vinc], which were all set to 1 *μ*M, with [vinc] being constant throughout the simulation (see Methods).

The baseline signal decay parameters (Table A in S1 Appendix) were set to match experimentally measured time periods for the assembly phase of NAs [17], leading to the concentration of signal crossing the *signal_thresh_* at *t_sig_* = 58.04 s (Fig 3A, Fig C and Text B in S1 Appendix). Additionally, when maturation (actin-binding) reactions were disabled, the predicted concentration of integrins in seeds and clusts was ≥ 0.1 *μ*M (Fig 3A) for approximately 158 s, a duration indicative of the lifetime of nascent adhesions and is in line with experimentally measured average lifetimes of NAs of 135–180 s [16, 17, 47]. Additionally, the concentrations of [S1] and [C1] also matched experimentally observed trends in abundance of early NAs [17] (Fig 3A)

When actin-binding reactions were allowed however, the concentration of [S1a] and [C1a] reached a peak at 58 s (Fig 3B), followed by a sharp fall to 0. This decrease is because the signal-dependent reduction in actin-binding rates reduced the formation of these species, but the rate constants (*k*_7*f*_ and *k*_12*f*_) of reinforcement reactions, Rx7 and Rx12, that transform S1a and C1a to S2a and C2a, respectively, remained unchanged. Thus, S1a and C1a were almost completely consumed after approximately 158 s. The predicted concentrations of [S1a] and [S3a] are highest on *k_sub_* = 100 pN/nm and *k_sub_* = 1 pN/nm substrates respectively (Fig 3B), whereas [C1a] and [C3a] are always higher on *k_sub_* = 1 pN/nm. These differences arise from the stiffness-dependent reinforcement rates, and the reversible mass-action kinetics considered here. This is explained in more detail in Text B in S1 Appendix. As such, the model accounts for one cycle of NA assembly, followed by either maturation or disassembly. When maturation was enabled, the concentrations of [S2a], [C2a], [S3a] and[C3a] reach a steady state after 600 s, in that they oscillate between 0 and an almost constant peak concentration (Fig 3B). In summary, the balance between the reversible mass-action kinetics, stiffness-dependent reinforcement rates, and signal concentration decay results in the formation and subsequent disassembly of low-order species (S1, C1, S1a, C1a) representing NAs, while mid- and high-order species (S2, C2, S2a, C2a, S3, C3, S3a, C3a) represent stable adhesions that may further mature to become FAs.

### Adhesion maturation is highest on moderate substrate stiffness

The concentrations of [S3a], [C2a] and [C3a] are highest on a moderate substrate stiffness (1 pN/nm), and lower on stiffer or softer substrates (Fig 3B) in accordance with experimental findings [4, 34, 56, 57]. This is a result of the time taken for the force on a clutch to reach the bond-rupture threshold being roughly equal to the lifetime of an unloaded AB-clutch that spontaneously dissociates from actin (or the substrate) due to thermodynamic fluctuations [4, 58]. Thus, the lifetime of a complete ECM-integrin-adaptor protein actin chain is maximized, resulting in more maturation. In addition, in the early periods of the simulations (0 to 70 s, Fig D in S1 Appendix), the concentrations [C3a], [C2a] and [S3a] increase most rapidly on *k_sub_*=1 pN/nm. While these results are for simulations with a constant vinculin concentration, similar results were obtained for limited vinculin conditions (see Text B and Fig E in S1 Appendix).

Although the concentration plots in Fig 3B are oscillatory due to the repeated bond-rupture events that transform AB clutches to AUB clutches, pushing the concentration of AB clutches to 0 and causing a spike in the concentration of AUB clutches, the peaks approach a steady state. We observed generally shorter periods of oscillations for AB clutches on stiffer substrates (Table 2), which is also reported by Venturini and Saez (2023) [38]. The periods predicted in our simulations were in good agreement with previous studies (Table 2) [4, 38, 58, 59]. Note that the periods for C2a and C3a on *k_sub_* = 0.1 pN/nm, are of the order of the lifetime of NAs (∼60 s) or higher. Thus, these results suggest that C2a and C3a can represent (partially) mature adhesions and not NAs, and that adhesions are likely to disassemble before sufficient reinforcement can occur on very soft substrates.

**Table 2:** Mean periods in s of different actin-bound clutches for different substrate stiffnesses.

Substrate stiffness (pN/nm)	S1a	S2a	S3a	C1a	C2a	C3a
0.1	15.37	32.72	55.74	30.43	64.83	110.48
1	2.01	4.22	7.15	3.67	7.76	13.18
10	0.47	0.94	1.58	0.62	1.26	2.13
100	0.31	0.61	1.02	0.33	0.64	1.07

In essence, after the initial NA formation phase, the number of mature adhesions grows, most rapidly on a moderate substrate stiffness of *k_sub_* = 1 pN/nm, and approaches a steady state. While the number of adhesions actively bound to the actin network constantly fluctuates due to force thresholds being reached or stochastic bond-rupture events, on average the number approaches a stable steady state.

### Traction force is highest on substrates of moderate stiffness

**Fig 4:**
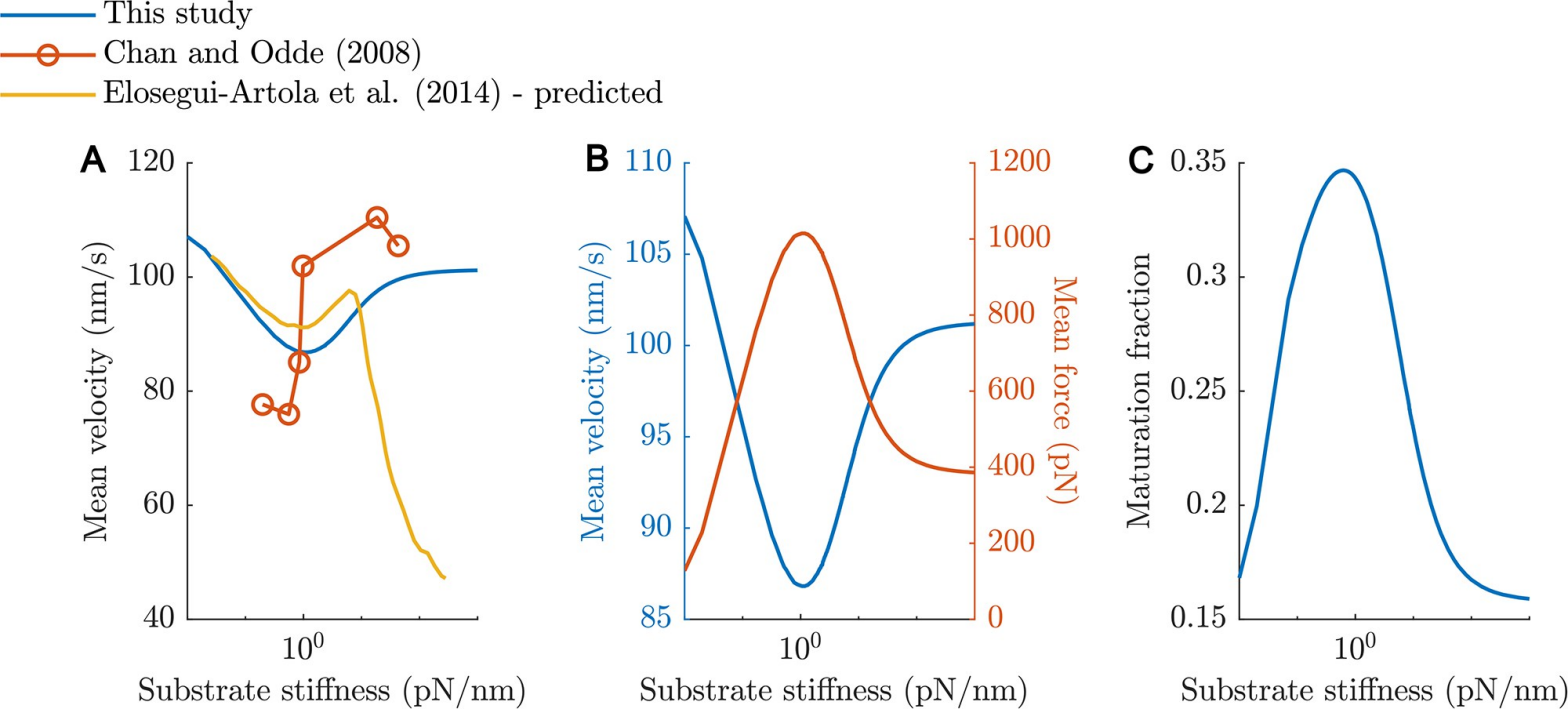
Model predictions of mean actin retrograde velocity and maturation fraction for the baseline model. (A) shows the predicted velocity vs substrate stiffness compared to previous studies, (B) shows the predicted velocity (blue) and mean force exerted by all adhesions (red) in this model, and (C) shows the NA maturation fraction vs substrate stiffness.

An optimal substrate stiffness is one at which the highest traction force is generated at the adhesions [60]. As explained in the previous section, this arises due to a balance between the lifetime of an unloaded AB clutch and the time taken for a clutch to reach the force threshold. In our simulations, the highest traction force was reached at *k_sub_* = 1 pN/nm, which also corresponded to the point where the lowest retrograde velocity was recorded (Fig 4A). The frequency of oscillations was higher for stiffer substrates (Table 2) given that the force on clutches increases faster on stiff substrates, thus reaching the force thresholds earlier, causing bond-rupture, and subsequent rebinding of the AUB-clutches to actin. While on softer substrates the force build-up is much slower as the substrate is more compliant (hence the lower oscillation frequency), actin unbinding due to thermodynamic fluctuations dominates as bonds break spontaneously much before the force thresholds are reached, giving rise to a higher actin unbinding rate (Fig F in S1 Appendix). These results suggest that *k_sub_* = 1 pN/nm gives rise to a ‘load-and-fail’ regime where clutches are loaded at a moderate rate, reach their force thresholds and subsequently break, ‘frictional slippage’ occurs on stiffer substrates where rapid loading causes clutches to disengage too quickly, resulting in lower average AB-clutch concentration [4, 60]. Altogether, these observations show that the optimal stiffness for NA maturation in our model is at *k_sub_* = 1 pN/nm.

Fig 4A shows the agreement between the mean retrograde velocity in our simulations and other computational [36, 38] and experimental [4] studies. A recent computational study also reports a similar biphasic behaviour with an optimal stiffness of around 10 pN/nm [38]. However, this is only observed when the weakest link in the force chain in their model is simulated as a catch bond. Additionally, our model predicts a linear increase in the mean velocity between 10^0^ pN/nm and 10^2^ pN/nm (Fig 4B), which is the stiffness range where the cell is mechanosensitive—a change in stiffness translates linearly into a change in actin retrograde velocity. This is in good agreement with previous studies which report ranges of 10^0^–10^1^ to 10^0^–10^2^ pN/nm [4, 57, 61]. The decrease in the predicted retrograde velocity for stiffnesses > 10^1^ pN/nm in the computational study of Elosegui-Artola et al. [36] (Fig 4A) arises because of reinforcement which they model as an increase in integrin density beyond a certain threshold force on a clutch. While the range of velocities reported varies, in our model, the lowest velocity depends on the concentration of myosin motors *conc_myo_*, which is a free parameter that was adjusted such that the lowest velocity was within 10% of that reported by Chan and Odde (2008) [4].

It is also important to note that the optimal stiffness of 1 pN/nm is reached only when the TDRM of actin-unbinding rates was applied (see subsection ‘Force-dependent actin-unbinding and time-dependent rate modification (TDRM)’ in Methods and Text A in S1 Appendix). For high substrate stiffnesses, since the time taken to reach the force thresholds is short, maturation is limited. For soft stiffnesses, however, this is not the case. Hence, in the absence of TDRM, a method we use to account for the force-independent spontaneous bond ruptures, the equilibrium concentrations of mid- and high-order AB clutches ([S2a], [S3a], [C2a], [C3a]) are highest on *k_sub_* = 0.1 pN/nm and decrease monotonically with increasing substrate stiffness (Fig G in S1 Appendix). Importantly, the decrease in equilibrium concentrations of mid- and high-order AB clutches caused by TDRM is the largest on *k_sub_* = 0.1 pN/nm and least on *k_sub_* = 100 pN/nm (Figs G.A and G.B in S1 Appendix). Thus, TDRM of disassembly rates is essential for obtaining an optimal stiffness through mechanosensing. As adhesion assembly and disassembly are tightly regulated processes, these results suggest altering factors affecting adhesion disassembly allows for more robustness and resilience in the mechanosensing and adhesion maturation processes.

### Predicted NA maturation fraction is most sensitive to talin stiffness and vinculin availability

After identifying species in the model that represent NAs (S1, C1, S1a, C1a) and adhesions that mature to FAs (S2, C2, S2a, C2a, S3, C3, S3a, C3a) based on comparisons of their concentrations, bond formation and rupture times to values reported in literature, we used our model to predict the fraction of NAs that may mature into FAs on a range of substrate stiffnesses. Notably, our model also predicts a biphasic trend in maturation fraction (MF) (Fig 4C). More specifically, the MF ranges from approximately 18% on very soft substrates (10^-2^ pN/nm) to around 34.3% on the optimal substrate stiffness of 10^0^ pN/nm, which lies within experimentally determined ranges of MFs under different conditions [18, 62].

To ensure that both the optimal stiffness and MF predictions were not heavily influenced by the choice of parameter values, we performed a local sensitivity analysis on 21 parameters (Text B, Figs I and J in S1 Appendix), and address the most important and representative ones here.

**Fig 5:**
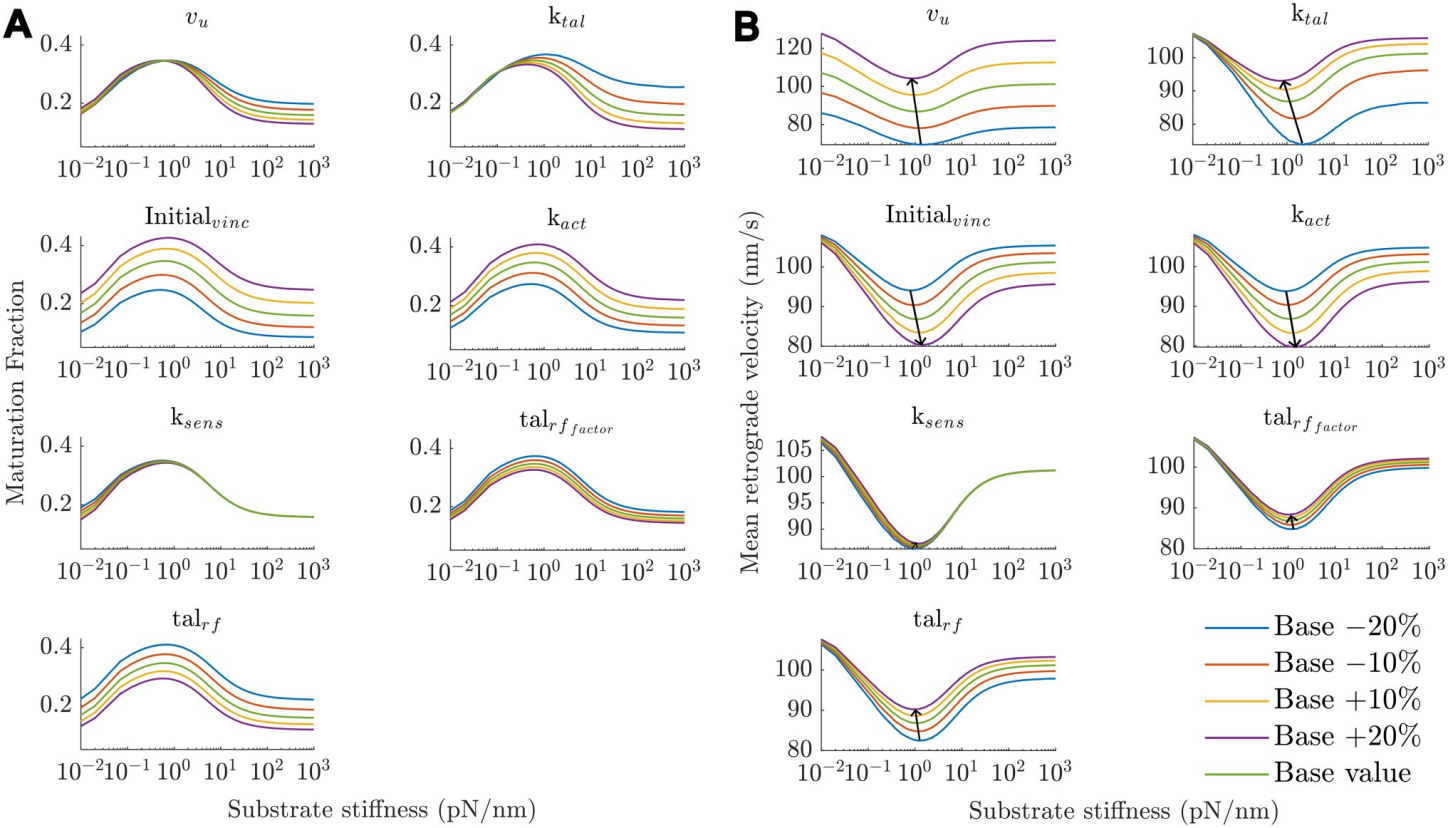
Sensitivity analysis results. (A)—Maturation fraction vs stiffness (outcome 1) and (B)—Mean retrograde velocity vs stiffness for a local variation in parameter values of different parameters. Black arrows in (B) point in the direction of increasing parameter value and track the optimal stiffness (outcome 2).

The optimal substrate stiffness, the stiffness at which the lowest mean retrograde velocity is observed (Fig 5B), was most sensitive to changes in *k_tal_*, the stiffness of talin. Increasing *k_tal_* shifts the optimum stiffness to softer substrates and reduces the MF (Fig 5B). Talin is the most abundant mechanosensitive component in the model and majorly contributes to determining the stiffness of clutches, which effectively determines the optimum substrate stiffness. Increasing the stiffness of talin results in stiffer clutches that reach the bond force thresholds sooner, leaving less time for maturation reactions and consequently lower MF.

**Fig 6:**
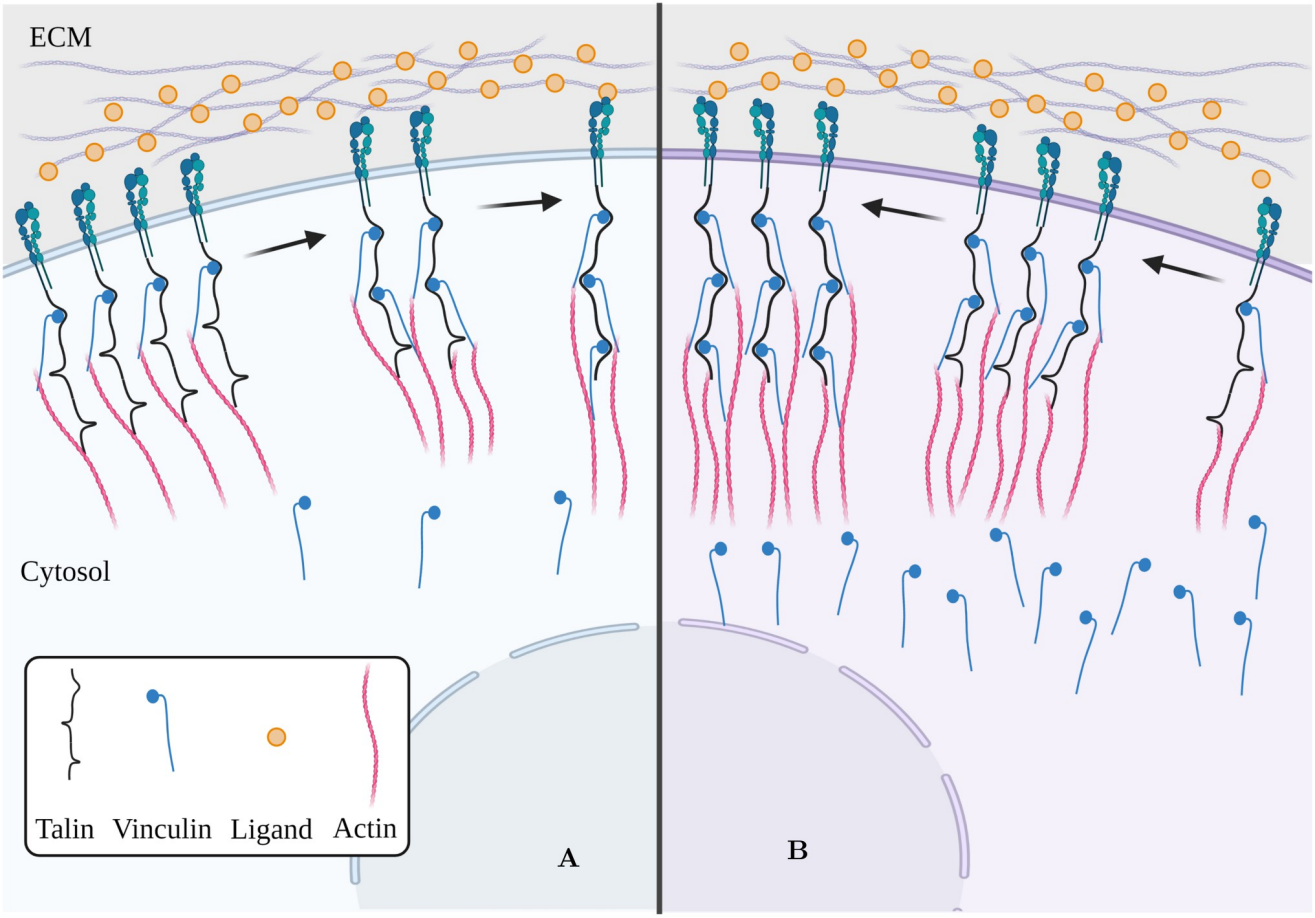
Vinculin concentration can influence maturation fraction. (A) and (B) are cell types or biological contexts where vinculin availability is low and high respectively. In A, the likelihood of vinculin binding to the exposed vinculin-binding sites on talin is low leading to a low maturation fraction. However, in B, due to a relatively higher vinculin availability, the integrin-actin link is highly likely to be reinforced by vinculin, increasing the maturation fraction. This figure was created using BioRender.com.

Increases in initial vinculin concentration *Initial_vinc_* leads to large increases in MF (Fig 5A) and small increases in the optimal stiffness (Fig 5B). A higher vinculin concentration increases the likelihood of maturation leading to increased force-carrying capacity and consequently a shift of the optimal stiffness to stiffer regimes. In contrast, a lower vinculin availability leads to decreased maturation fractions and traction force and a higher mean retrograde velocity (Figs 5 and 6).

Changes in the talin refolding rate, *tal_rf_*, affect the mean retrograde velocities more than the optimum stiffness (Fig 5). A higher *tal_rf_* leads to lower maturation fractions but has negligible effects on the optimum stiffness. This is because a higher talin refolding rate causes AUB clutches unbinding from vinculin more likely and hence lowers the concentration of more mature, higher force-carrying-capacity clutches on all substrate stiffnesses. This, however, does not cause any changes in the relative proportions of concentrations of different clutches across substrate stiffnesses and hence does not change the optimum stiffness considerably. A similar reasoning is valid for a lower *tal_rf_*.

The talin refolding factor, ${tal}_{{rf}_{factor}}$, controls the rate of vinculin-dissociation and hence relative additional stability of higher order species in our model (see Text A in S1 Appendix) and its baseline value is set to 0.5 in our simulations (talin in higher order species is half as likely to refold compared to mid-order species). Increasing ${tal}_{{rf}_{factor}}$ results in lower MFs and shifts the optimal stiffness to softer substrates due to the decreased ‘additional’ stability of higher-order species. Similar reasoning can be used to explain the effects of decreasing ${tal}_{{rf}_{factor}}$. As vinculin is known to ‘lock’ talin in the unfolded conformation [39], we further reduced the value of ${tal}_{{rf}_{factor}}$ to 0.2 to investigate the effects of having highly stable high-order species. This resulted in the same trends of maturation across different stiffnesses but with slightly higher maturation fractions (Fig H in S1 Appendix).

An increase in *v_u_*, the unloaded actin retrograde velocity, pushed the optimal substrate stiffness to softer substrates in line with previous computational studies [57, 60] and leads to lower MF (Fig 5). A higher retrograde velocity causes faster force build-up resulting in frictional slippage on softer substrates. Similar to *k_tal_*, it also results in lower MF. On the contrary, increases in *k_act_* pushed the optimal substrate stiffness towards stiffer substrates and increases MF (Fig 5), which is due to the ‘strengthening’ of clutches as they are more likely to bind actin, get stretched and recruit vinculin, and on average there are more AB clutches resulting in higher forces on softer substrates [57, 60].

Out of all the parameters, the stiffness of talin *k_tal_*, initial vinculin concentration *initial_vinc_*, talin refolding rate *tal_rf_*, and the actin-binding rate *k_act_* had the greatest influence on MF, similar for both an increase and decrease in the parameter values (Fig I in S1 Appendix). Importantly, the TDRM factor *k_sens_*, and the cluster formation (*k*_14*f*_, *k*_15*f*_, *k*_16*f*_) and disassembly (*k*_21*f*_, *k*_22*f*_) rates had negligible influences on the MF and optimal stiffness (Figs I and J in S1 Appendix) for the tested range of values (±20%).

## Discussion

Although cell-ECM adhesions are extensively studied, the effects of mechanical properties of the ECM and intracellular proteins on the early processes of adhesion assembly, maturation and traction force generation remain unclear. Here, we present a computational model that innovatively bridges the discrete mechanical and continuous biochemical aspects of adhesion formation. Our model captures key trends in the maturation fraction (MF) of NAs, actin retrograde velocity, and the periods of bond formation-rupture cycles, all in agreement with experimental evidence [4, 18, 59, 62]. The predicted optimal substrate stiffness [4] and stiffness sensitivity range [57, 61] also lie within experimentally determined ranges.

While the predicted mean actin retrograde velocity across the stiffness range tested is in agreement with an experimental study using embryonic chick forebrain neurons [4], the agreement with the study of Elosegui-Artola and colleagues (2014) [36] is limited to the softer regimes where the biphasic trend is also seen (compare yellow and blue lines in Fig 4A). This discrepancy arises from the way reinforcement of adhesions is modelled. In particular, reinforcement in that study is modelled as an increase in the integrin density that occurs if a clutch experiences a force ≥87 pN, leading to an increase in integrin–ECM binding events and a larger number of bound clutches. In a recent computational study where reinforcement is also modelled similarly, a biphasic behaviour is observed just as in our model but with the optimal stiffness being around 10 pN/nm [38]. In our model, while there is an increase in the cluster size of clutches and additional vinculin recruitment leading to larger force-carrying capacities, there is no change in the number of available integrins or the adhesion formation rates.

In this study, we assume relatively fast kinetics for the signal molecule to keep the NA assembly and disassembly phases in line with experimental data [17]. It is important to note that there may be considerable differences in experimental results based on the cell types used, resulting in different time scales. However, since the model is relatively insensitive to changes in *signal_thresh_* (Figs I and J in S1 Appendix), and consequently changes in *t_sig_*, the overall behaviour of the model is unlikely to change drastically when these parameters are tuned to represent specific cell types or, for instance, signal molecule kinetics. Thus, the generic signal molecule in the model can potentially represent the level of unphosphorylated FAK or similar molecules whose change in (phosphorylation) state can set off signalling cascades leading to adhesion disassembly. Future work should aim to determine the underlying factors that induce and influence adhesion disassembly so that the generic signal molecule can be replaced with more accurate formulations and interactions. In particular, the identification of such concrete factors could help in determining the settings of *signal_thresh_*.

Based on our results, the factors affecting the NA disassembly dynamics play a more important role than those affecting assembly dynamics. We applied TDRM, an innovative method to account for spontaneous bond-rupture events in NA formation in an ODE framework. TDRM was necessary to establish the optimal stiffness because in its absence, maturation is highest on soft substrates as forces on the clutches build up slowly, giving long durations for maturation reactions to occur. TDRM counters this by increasing the rate of clutch-actin bond rupture and hence prevents maturation. With the baseline value of the TDRM factor *k_sens_*, the effect of TDRM on the bond-rupture rate is highest on soft substrates and negligible on stiffer substrates due to the short clutch lifetimes. Walcott et al. (2011) [63] predicted and experimentally verified that disassembly processes begin earlier for soft substrates, and this arises from the force- and strain-dependent bond formation and rupture probabilities. In addition, cyclic mechanical reinforcement (CMR) of integrin-ligand bonds strengthens them, increasing the lifetimes, implying that on soft substrates where force-loading is relatively slow and force remains low for longer durations, these bonds are less reinforced and are more likely to break [53]. In previous studies, CMR has been modelled as an increase in bond-dissociation rates at low forces [35, 38]. TDRM can be considered as a method to coarsely account for these processes. However, while the outcomes of TDRM are similar to the effects of CMR as modelled in [35, 38], the differences between the two methods need to be investigated further. Surprisingly, the optimal stiffness was insensitive to changes in the parameter that controls the magnitude of TDRM (*k_sens_*, (Fig 5B). This was unexpected since TDRM was essential for establishing an optimal stiffness implying a major role of this parameter in determining model behaviour (Figs F and G in S1 Appendix). It is likely that the explored sensitivity range (±20%) was too narrow to considerably change the behaviour of the model, which should be investigated more in-depth in the future. Note as well that in this study we performed a local sensitivity analysis focusing on those parameters that can directly be traced to a biological phenomenon (for instance *k_act_*, *Initial_vinc_*), those that we introduced as part of SDRM and TDRM (for instance *k_sens_*, *signal_thresh_*), or assumed (for instance, ${tal}_{{rf}_{factor}}$). Considering the non-linear nature of the model, it would be interesting in future studies, to conduct a more rigorous, global sensitivity analysis (i.e. using Bayesian Optimization) to further identify the most significant parameters of the model.

Another benefit of our model is that it allows the prediction of the maturation fraction of adhesions for a range of substrate stiffnesses. While there are no studies to the best of our knowledge that explicitly investigate the MFs for different substrate stiffnesses, the predicted range of MFs for the stiffness range tested in this study was within the range of experimentally determined fractions [18, 62].

Our sensitivity analysis results show that the MF is highly influenced by the concentration of integrins and vinculin available, the actin-binding rate, and the talin-refolding rate (Fig 5A). These factors can possibly be experimentally controlled, by introducing mutations in the proteins, allowing the predictions to be tested. Additionally, the MF and optimal stiffness were found to be insensitive to variations in the TDRM factor *k_sens_* and the cluster formation and disassembly rates, suggesting that the model is locally robust to these factors and the parameters can be tuned to be specific to experimental conditions or cell lines. This suggests that the model can be used to predict the MFs for a variety of conditions by varying molecular stiffnesses, initial concentrations of talin, integrin, and vinculin, different clustering, maturation, and disassembly rates among many other parameters. This can potentially shed light on how traction force exerted by the cell is affected by biochemical alterations within the cell.

For instance, vinculin plays an important role in both cell-ECM adhesions and cell-cell adhesions through cadherins. Numerous studies indicate interdependence and cooperativity of these two processes, mediated through signal cascades or proteins that are essential in both types of adhesions, to varying degrees in different cell types [64–69]. While vinculin knockout studies have shown that traction force generation is impaired, with some studies reporting a decrease of nearly 50% in the absence of vinculin, overexpression of vinculin results in extremely strong adhesions that suppresses cell motility [70–75]. However, this leaves unanswered questions about how relatively less drastic changes in vinculin availability arising from cross-talk between integrins and cadherins adhesion complexes affect traction force generation and adhesion maturation. Our model predicts that a 20% decrease in the vinculin concentration results in a ∼9% increase in the actin retrograde velocity (or equivalently a 9% decrease in the traction force exerted due to lower MFs) at the optimal substrate stiffness (Fig 5). Thus, our model can be especially valuable to make predictions and generate hypotheses about how (local) adhesion protein concentrations influence the early processes of adhesion assembly, maturation, and traction force generation.

Overall, our model improves on previous studies in several aspects. Firstly, the process of maturation is more accurately captured by accounting for multiple vinculin recruitment events that progressively increase the clutch stiffness in a continuous ODE framework. Previous studies either did not account for this or at most accounted for recruitment of one vinculin [4, 30–35]. Secondly, this model couples changes in discrete mechanical factors of adhesion maturation such as clutch stiffness with the continuous framework of biochemical reactions underlying adhesion maturation. This is particularly important because the continuous biochemical models do not explicitly account for force on the clutches, and discrete mechanical models of adhesion formation do not capture the resulting experimentally measurable biochemical changes that occur.

While it is clear from our results that the adhesion assembly and disassembly rates must be dynamic and dependent on a signal to achieve the maturation of only a fraction of the NAs that are initially formed, we acknowledge several limitations to this study. First, we do not model numerous proteins involved in the process of maturation or the continuous increase in the area of the adhesion [76]. Second, we simplified vinculin recruitment and growth of cluster size to occur in two discrete steps, and no spatial effects (e.g. proximity to an actin fibre, the distance of adhesion from the cell membrane) are accounted for. And third, we assume that integrins, talin and vinculin are available in roughly equal proportions near the adhesions (initial concentrations are the same), which may not necessarily be true. Despite these limitations, our model reproduced experimentally observed trends with respect to force, substrate stiffness, and time periods of oscillation in concentrations of the different seeds and clusts [4, 59, 60]. Furthermore, our results are reasonably close to discrete, stochastic computational studies as mentioned earlier even though our model bridges discrete and continuous aspects. The model thus provides a reliable foundation for further investigations.

What remains to be explored, perhaps by building on our model, is the interaction between the various signal cascades that regulate NA maturation. The ubiquitous signaling molecule FAK is also force-activated adding a further layer of interactions and complexity [77, 78]. In addition, the KANK family of proteins are known to impair the actin-binding capacity of talin, thereby weakening the integrin-actin linkage, and affecting the catch and slip bond dynamics [79]. They also play a role in targeting microtubules to focal adhesions which aids in their disassembly through multiple signal cascades [80]. By expanding the current model framework to include these interactions, it has the potential to robustly simulate the mechanochemical processes underlying mechanotransduction and provide valuable insight into cell signalling, communication and organization, hence contributing to advances in developmental biology and regenerative medicine.

### Implementation

The model and the simulations were implemented using MATLAB R2020a [81]. All code and scripts used in this study are publicly available via GitHub at https://github.com/CarlierComputationalLab/force-dependent-adhesion-composition.git.

## Supporting information

S1 Appendix Detailed explanations of the methods (Text A), additional results (Text B), figures (Fig A—Fig N) and tables (Table A and Table B). [83–104] are cited in this file.
(ZIP)
