# Supplementary material for: Force-dependent focal adhesion assembly and disassembly: A computational study
Source: PLoS Comput Biol. 2023 Oct 6;19(10):e1011500. doi: 10.1371/journal.pcbi.1011500 (PMC10584152; doi:10.1371/journal.pcbi.1011500)
Supplement: S1 Appendix — Detailed explanations of the methods (Text A), additional results (Text B), figures (Fig A—Fig N) and tables (Table A and Table B). [83–104] are cited in this file. (ZIP) [file pcbi.1011500.s001.zip › S1_Appendix.pdf]

Supporting information - Force-dependent focal adhesion assembly and disassembly: a computational study

Kailas Shankar Honasoge[1☯], Zeynep Karagöz[1☯], Benjamin T. Goult[2], Haguy Wolfenson[3], Vanessa. L. S. LaPointe[1], Aurélie Carlier[1*]

1 Department of Cell Biology–Inspired Tissue Engineering, MERLN Institute for Technology-Inspired Regenerative Medicine, Maastricht University, Maastricht, the Netherlands
2 School of Biosciences, University of Kent, Canterbury, UK
3 Department of Genetics and Developmental Biology, Rappaport Faculty of Medicine, Technion – Israel Institute of Technology, Haifa, Israel

☯Shared first author.
* a.carlier@maastrichtuniversity.nl

S1 Appendix

Text A. Methods

Hookean spring system The substrate–integrin–adaptor protein system was formulated as a system of Hookean springs. As seeds and clusts contain 25 and 50 integrin–adaptor protein complexes connected in parallel (Fig 1C, main text), their stiffnesses are 25 and 50 times the stiffness of a single integrin–adaptor protein complex (IAPC). This is derived in the following section.

Stiffness of an integrin-adaptor protein complex Talin has multiple hidden vinculin binding sites that are uncovered as the talin rod unfolds due to the application of force in the process of reinforcing the link between the ECM and the actomyosin network of the cell. Talin was found to form a pre-complex with one vinculin molecule before entering adhesions [1]. Thus, the fundamental building block of this model is an integrin–talin–vinculin complex as seen in Fig A. On application of force and experiencing a stretch, up to two more vinculin molecules are allowed to bind to represent two reinforcement steps. The vinculin binding sites are assumed to be evenly spaced on the talin rod. Thus, the stiffness of a complex containing one talin rod and n_{vinc} vinculin molecules is described in the following way.

Generally, the combined stiffness $k_{combined}$ of two springs with stiffnesses of $k_{spring1}$ and $k_{spring2}$ connected in series is given by

$$k_{combined} = \left(\frac{1}{k_{spring1}} + \frac{1}{k_{spring2}}\right)^{-1} \tag{S1}$$

If connected in parallel, the combined stiffness is

$$k_{combined} = k_{spring1} + k_{spring2} \tag{S2}$$

Let k_{tal} be the stiffness of talin, k_{vinc} be the stiffness of vinculin, and k_{sub} be the stiffness of the substrate (ECM). Fig A shows a generalised integrin–adaptor protein complex bound to the substrate. The talin rod can be represented as four sub-springs of

equal stiffness such that the stiffness when they are all connected in series is equal to k_{tal}. Namely springs A, B, C, and D represent the four sub-segments such that $k_{tal} = 4{\cdot}k_{4tal}$, where k_{4tal} is the stiffness of a sub-segment.

$$k_{tal} = \left(4{\cdot}\frac{1}{k_{4tal}}\right)^{-1} \rightarrow k_{4tal} = 4{\cdot}k_{tal} \tag{S3}$$

Vinculin molecules are represented by springs E, F, and G. Using Eq. S1 and Eq. S2, the effective stiffness of the system of springs containing springs A − G is given by:

$$k_{complex} = \left(\frac{1}{A} + \frac{1}{E + \left(\frac{1}{F+\left(\frac{1}{D+G}+\frac{1}{C}\right)^{-1}} + \frac{1}{B}\right)^{-1}}\right)^{-1} \tag{S4}$$

The stiffness of springs A, B, C, and D is k_{4tal} (Eq. S3). The stiffness of springs E, F, and G is k_{vinc} (0.25 pN/nm [2]). Note that for complexes with one vinculin, G and F is 0, and similarly for complexes with two vinculins, G is set to 0.

Fractional extension of clutches When talin binds to actin, the chain of links between the cell and the substrate is complete. Due to actin retrograde velocity of v_u, this system of springs containing the spring representing the substrate connected in series to a spring that represents the clutch (seed or clust) experiences a total extension of $v_u{\cdot}dt$, where dt is the time step. This extension is thus shared between the substrate and the clutch based on their relative stiffnesses. The fraction of this extension experienced by the clutch is given by a_{clutch} is calculated in the following way.

Let k_{clutch} be the stiffness of the clutch ($25{\cdot}k_{complex}$ for a seed, $50{\cdot}k_{complex}$ for a clust) and k_{sub} be the stiffness of the substrate. The integrin is considered to be a massless rigid body that does not undergo any physical change on the application of force. Thus, the system of springs is then reduced to two springs of stiffnesses k_{clutch} and k_{sub} connected in series, which can be represented by a 'combined' spring *comb* with an effective stiffness k_{comb} given by

$$k_{comb} = \left(\frac{1}{k_{clutch}} + \frac{1}{k_{sub}}\right)^{-1} = \frac{k_{clutch}{\cdot}k_{sub}}{k_{clutch} + k_{sub}} \tag{S5}$$

Thus, this spring experiences an extension of $v_u{\cdot}dt$ in every time step. The force on this spring is then,

$$F_{comb} = k_{comb}{\cdot}\left(x_{comb}\right) \tag{S6}$$

where $x_{comb} = v_u{\cdot}dt$.

Since the extension x_c is the sum of extensions of the springs representing the substrate and the clutch,

$$x_{comb} = x_{sub} + x_{clutch} \tag{S7}$$

where x_{sub} and x_c are extensions of the substrate and clutch springs respectively.

Therefore,

$$x_{clutch} = x_{comb} - x_{sub} \tag{S8}$$

Since springs in series experience the same force, the force on the substrate spring is equal to the force on the *comb* spring. Therefore

$$x_{clutch} = x_{comb} - \frac{F_{sub}}{k_{sub}} = x_{comb} - \frac{F_{comb}}{k_{sub}} = x_{comb} - \frac{k_{comb} \cdot x_{comb}}{k_{sub}} \tag{S9}$$

This implies

$$x_{clutch} = x_{comb} \cdot \left(1 - \frac{k_{comb}}{k_{sub}}\right) = x_{comb} \cdot \left(1 - \frac{k_{clutch}}{k_{clutch} + k_{sub}}\right) \tag{S10}$$

$$\rightarrow \; x_{clutch} = x_{comb} \cdot \left(1 - \frac{k_{clutch}}{k_{clutch} + k_{sub}}\right) = x_{comb} \cdot a_{clutch} \tag{S11}$$

where $a_{clutch} = \left(1 - \frac{k_{clutch}}{k_{clutch}+k_{sub}}\right)$ is the fractional extension of a clutch of stiffness k_{clutch} is connected to a substrate of stiffness k_{sub}.

Force on the fourth talin sub-spring The talin–actin bond is a 2 pN slip bond [3]. However, integrins bound to talin that is further bound to multiple vinculins can carry a force much higher than 2 pN [4]. For both of these to be true, the different domains of the talin rod must experience different forces, and the talin–actin slip bond is likely dependent on the force experienced by the talin rod domain that binds actin. Here, the fourth talin sub-spring binds actin and hence the slip bond rupture occurs when this particular spring experiences a force of 2 pN.

The force experienced by spring D in Fig A is given by $F_D = k_D \cdot x_D$ where $k_D = k_{4tal}$, and x_D is the extension of spring D. x_D in terms of the extension of the entire system x_{comb} (i.e. the extension of the *comb* spring) is derived as follows. Note that x_{ijk} is the general representation of the extension of the spring system containing springs i, j, and k.

The total force on the system:

$$F_{comb} = k_{comb} \cdot x_{comb} \tag{S12}$$

Since springs in series experience the same force,

$$F_{comb} = F_{sub} = F_{clutch} = F_A \tag{S13}$$

Extension of clutch:

$$x_{clutch} = x_{comb} - x_{sub} \tag{S14}$$

Extension of spring A:

$$x_A = \frac{F_A}{k_A} = \frac{F_{clutch}}{k_A} = \frac{k_{clutch} \cdot x_{clutch}}{k_A} \tag{S15}$$

Extension of spring system containing springs B – C – D – F – G:

$$x_{BCDFG} = x_{clutch} - x_A \tag{S16}$$

Since springs in series experience the same force, force on spring B:

$$F_B = F_{BCDGF} \tag{S17}$$

Extension of spring B:

$$x_B = \frac{F_B}{k_B} = \frac{F_{BCDFG}}{k_B} = \frac{k_{BCDFG} \cdot x_{BCDFG}}{k_B} \tag{S18}$$

Extension of spring system containing springs C – D – G:

$$x_{CDG} = x_{BCDFG} - x_B \tag{S19}$$

Since springs in series experience the same force, force on spring C:

$$F_C = F_{CDG} \tag{S20}$$

Extension of spring C:

$$x_C = \frac{F_C}{k_C} = \frac{F_{CDG}}{k_C} = \frac{(k_{CDG} \cdot x_{CDG})}{k_C} \tag{S21}$$

Extension of spring D:

$$x_D = x_{CDG} - x_C \tag{S22}$$

On simply resubstituting for x_c and x_{CDG} in Eq. S22 using Eq. S12 to S21, we obtain:

$$x_D = (x_{clutch} - x_A) \cdot \left(1 - \frac{k_{BCDFG}}{k_B}\right) \cdot \left(1 - \frac{k_{CDG}}{k_C}\right) \tag{S23}$$

Since $x_{clutch} = x_{comb} \cdot a_{clutch}$, we get

$$x_D = (x_{comb} \cdot a_{clutch} - x_A) \cdot \left(1 - \frac{k_{BCDFG}}{k_B}\right) \cdot \left(1 - \frac{k_{CDG}}{k_C}\right) \tag{S24}$$

From Eq. S13 we have $F_A = F_{clutch}$. Hence,

$$x_A = \frac{F_A}{k_A} = \frac{F_{clutch}}{k_A} = \frac{k_{clutch} \cdot x_{clutch}}{k_A} = \frac{k_{clutch} \cdot (x_{comb} \cdot a_{clutch})}{k_A} \tag{S25}$$

Therefore, the extension of spring D is given by

$$x_D = (x_{comb} \cdot a_{clutch}) \cdot \left(1 - \frac{k_{clutch}}{k_A}\right) \cdot \left(1 - \frac{k_{BCDFG}}{k_B}\right) \cdot \left(1 - \frac{k_{CDG}}{k_C}\right) \tag{S26}$$

Reactions in the model

Integrin activation Integrins transition from a low-affinity to a high-affinity state when they bind to ECM ligands or cytosolic adaptor proteins such as talin [5, 6]. Once ligand-bound, the integrin is anchored to the ECM, enabling bi-directional transmission of force. In this study, we model $\alpha_5\beta_1$ integrins and assume they are activated.

Pre-complexation of integrin, talin and vinculin

$$Rx1 = k_{1f} \cdot [int] \cdot [tal] \cdot [vinc] - k_{1r} \cdot [Pcomp] \tag{S27}$$

Pre-complexation of talin and vinculin is necessary for NA maturation [1]. The first reaction is the combined reaction involving talin (tal) and vinculin ($vinc$) binding to integrin (int) to form an integrin-adaptor protein complex termed Pcomp. This species does not bind to actin and hence does not experience force. The reaction is reversible and has a forward rate constant of k_{1f} and a reverse rate constant of k_{1r}.

Integrin clustering Integrin clustering to form NAs is critical for cellular mechanosensitivity [7]. Clustering is initiated by integrin binding to talin [8], vinculin recruitment to the adhesion [9] and force application [10–12]. Ligand type and density on the ECM also influence clustering, with an upper limit for ligand spacing of around 60 nm [3, 10, 13]. Integrin clustering is a continuous process and clusters can grow to contain up to 50 integrins [12].

As a simplification, here the growth of clusters to the maximal considered size (50 integrin–adaptor protein complexes) is split into two stages with the initial clustering resulting in a small cluster of 25 integrin–adaptor protein complexes, termed 'seed' and denoted by 'Sx', and a second clustering reaction where seeds dimerize to give a large cluster with 50 integrin-adaptor protein complexes, termed 'clust' and denoted by 'Cx' (Fig 1C). Here, $x \in \{1, 2, 3\}$ and denotes the number of vinculin molecules in the individual integrin-adaptor protein complexes. Actin-bound seeds and clusts are denoted by 'Sxa' and 'Cxa' respectively.

Seed formation

$$Rx2 = k_{2f} \cdot [Pcomp]^2 - k_{2r} \cdot [S1] \tag{S28}$$

To form a seed, 25 Pcomps come together at a constant rate of k_{2f} and are assumed to bind simultaneously and parallelly to form the lowest order AUB seed, S1. S1 seeds breakdown to give 25 Pcomps at a constant rate of k_{2r}. The order of the reaction with respect to Pcomp is set to two to account for any intermediate steps that might be present in the process of clustering (setting the order to one gives similar results, see Fig L.

Clust formation through seed dimerization

$$Rx3 = k_{3f} \cdot [S1]^2 - k_{3r} \cdot [C1] \tag{S29}$$
$$Rx14 = k_{14f} \cdot [S1a]^2 - k_{14r} \cdot [C1a] \tag{S30}$$
$$Rx15 = k_{15f} \cdot [S2a]^2 - k_{15r} \cdot [C2a] \tag{S31}$$
$$Rx16 = k_{16f} \cdot [S3a]^2 - k_{16r} \cdot [C3a] \tag{S32}$$

S1 seeds reversibly dimerize with constant forward and reverse rates (k_{3f} and k_{3r}), forming lowest order AUB clusts, C1, containing 50 Pcomps. Higher-order AUB seeds (S2, S3) are not allowed to dimerize as they experience talin refolding [14, 15] and hence should be more likely to break down than to form clusts. However, AB seeds of all orders (S1a, S2a, and S3a) can dimerize to form AB clusts (C1a, C2a, and C3a).

Actin binding/unbinding

$$Rx4 = k_{4f} \cdot [S1] - k_{4r} \cdot [S1a] \tag{S33}$$
$$Rx5 = k_{5f} \cdot [S2] - k_{5r} \cdot [S2a] \tag{S34}$$
$$Rx6 = k_{6f} \cdot [S3] - k_{6r} \cdot [S3a] \tag{S35}$$
$$Rx9 = k_{9f} \cdot [C1] - k_{9r} \cdot [C1a] \tag{S36}$$
$$Rx10 = k_{10f} \cdot [C2] - k_{10r} \cdot [C2a] \tag{S37}$$
$$Rx11 = k_{11f} \cdot [C3] - k_{11r} \cdot [C3a] \tag{S38}$$

Actin-unbound (AUB) clutches (S1, S2, S3, C1, C2, C3) can bind to actin filaments through the actin-binding sites on talin and vinculin. This results in actin-bound (AB) clutch (S1a, S2a, S3a, C1a, C2a, C3a) (Fig 1C) that can experience and transmit force. The baseline actin-binding rate is k_{act} for all actin-binding reactions.

Actin-bound (AB) clutches can experience bond rupture either at the integrin–ECM catch-slip bond or the talin–actin slip bond as explained in the main text (section 'Force-dependent actin-unbinding and time-dependent rate modification (TDRM)') and supplementary sections 'Slip and catch bonds' and 'Time-dependent rate modification (TDRM)'. The combined bond-rupture rate of the catch-slip and slip bonds depend on the force on the clutches and is given by S66.

Adhesion reinforcement When talin is subject to extensions, a maximum of eleven vinculin-binding sites (VBS) that are otherwise cryptic are uncovered [16, 17]. This allows for more vinculin to be recruited to reinforce the bond with the actin network. In this model, we consider two vinculin-reinforcement events.

Since we assume vinculin binds instantaneously once the vinculin binding site is uncovered, the rates of the reinforcement reactions are equal to the rate at which talin unfolds, which depends on the force experienced by the clutch as explained in the supplementary section 'Force quantification'.

First vinculin reinforcement

$$Rx7 = k_{7f} \cdot [S1a] \cdot [vinc]^2 - k_{7r} \cdot [S2a] \tag{S39}$$

$$Rx12 = k_{12f} \cdot [C1a] \cdot [vinc]^2 - k_{12r} \cdot [C2a] \tag{S40}$$

A low order AB clutch (S1a, C1a) is stretched when it is pulled by actin. The first VBS is uncovered when each complex within the AB clutch experiences a force of 5 pN leading to additional vinculin binding [15]. The forward rates (k_{7f}, k_{12f}) of these reactions are dependent on the force experienced by the AB clutches undergoing reinforcement and are given by eq. 20 (main text).

Second vinculin reinforcement

$$Rx8 = k_{8f} \cdot S2a \cdot [vinc]^2 - k_{8r} \cdot [S3a] \tag{S41}$$

$$Rx13 = k_{13f} \cdot C2a \cdot [vinc]^2 - k_{13r} \cdot [C3a] \tag{S42}$$

A mid-order (S2a, C2a) AB clutch is stretched further on application of force resulting in a second VBS being uncovered when the force reaches 12 pN [16], allowing further vinculin binding. The forward rates of these reactions (k_{8f}, k_{13f}) depend on the force experienced by the AB clutches undergoing second reinforcement and are given by eq. 20 (main text).

Reinforcement of seeds and clusts is modelled as a single-step reaction where simultaneous recruitment of 25 and 50 vinculin molecules respectively occurs. However, the orders of the reinforcement reactions (Rx7, Rx8, Rx12, Rx13, Table B) with respect to vinculin were chosen to be 2 to approximately account for the influence of possible intermediate stages in the reactions.

Adhesion disassembly In the absence of sufficient force, adhesions disassemble because of mechanical and chemical signals [18]. Here, we model two parallel processes of disassembly, namely talin refolding and cluster breakdown.

Talin refolding

$$Rx17 = k_{17f} \cdot [S3] \tag{S43}$$

$$Rx18 = k_{18f} \cdot [S2] \tag{S44}$$

$$Rx19 = k_{19f} \cdot [C3] \tag{S45}$$

$$Rx20 = k_{20f} \cdot [C2] \tag{S46}$$

Once a seed or a clust unbinds from actin due to bond rupture (supplementary section Actin binding/unbinding), the clutch is no longer held under force. This increases the likelihood of stretched talin refolding and the recruited vinculin molecules dissociating. However, vinculin binding stabilizes stretched talin and reduces refolding rates[14]. Additionally, the vinculin exchange rate is nearly 60% higher in NAs than in stable adhesions[19]. Therefore, the first talin-refolding event that results in a high to mid order transition (S3 to S2 and C3 to C2, rate constants k_{17f}, k_{19f}) occurs at a slower rate than the second talin-refolding event that results in a mid to low order transition (S2 to S1 and C2 to C1, rate constants k_{18f}, k_{20f}). In particular, the rates of the first talin-refolding and second talin-refolding reactions are given by $k_{17f} = k_{19f} = tal_{rf_{factor}} \cdot tal_{rf}\ s^{-1}$ and $k_{18f} = k_{20f} = tal_{rf} \cdot sig_{dep}\ s^{-1}$ respectively, where $tal_{rf_{factor}}$ is the factor by which the first talin-refolding rate is slower than the second, and tal_{rf} is the baseline talin refolding rate (refer Table A). The talin-refolding events are irreversible reactions (Rx17, Rx18, Rx19, Rx20, Table B) since the VBS on talin is assumed to be covered for AUB seeds and clusts.

Clust breakdown to seed

$$Rx21 = k_{21f} \cdot [C3] \tag{S47}$$
$$Rx22 = k_{22f} \cdot [C2] \tag{S48}$$

AUB clusts can break down to give two AUB seeds through an irreversible reaction (Fig 1B). Since a high order clust (C3) is more robust to talin-refolding, we also assume it is more stable than a mid-order clust (C2). Hence, the high-order AUB clust to high-order AUB seed (C3 to S3) transition was assumed to be slower than mid-order AUB clust to mid-order AUB seed (C2 to S2). The baseline rates for these breakdown reactions were set at k_{21f}= 0.005 s^{-1} and k_{22f}=0.008 s^{-1}, respectively. While these values were arbitrarily chosen, they were of the same order of magnitude as reverse rates of seed dimerization reactions (Rx14, Rx15, and Rx16, Table B).

Actin retrograde velocity The cell membrane at the leading-edge pushes back on actin filaments undergoing actin-polymerization. Additionally, myosin II motors that are present away from the cell edge pull on the actin filaments with a force of 2 pN per motor [19–21]. These two forces cause an overall flow of actin filaments away from the cell membrane, known as the actin retrograde flow. Coupling the retrograde flow to the ECM through the adaptor protein–integrin–ligand chain effectively establishes a connection between the cell and its environment.

We assume that the force exerted by myosin II motors on actin filaments is balanced by the drag force arising due to the viscosity of the cytoplasm. Thus, in the absence of integrin-mediated forces on actin filaments, they move with a constant retrograde velocity. When clutches bind to the actin filaments, they provide resistance to the motion of actin filaments until they unbind, either spontaneously or because the catch/slip bond threshold is reached. This resistance decelerates the actin filaments. When the resistance provided by clutches is equal to the force exerted by myosin II motors, the actin filaments stop moving and the retrograde velocity is reduced to 0. Since all forces act along a single axis in the model, applying force balance gives a linear relationship between force and velocity:

$$v_{retro} = v_u \cdot \left(1 - \frac{F_{total}}{F_{myo}}\right) \tag{S49}$$

where v_u is the unloaded velocity of the actin filaments, F_{total} is the total force exerted by all AB clutches (see supplementary section 'Force quantification'), and F_{myo} is the total force exerted by myosin II motors.

F_{myo} is given by:

$$F_{myo} = n \cdot conc_{myo} \cdot F_{myo_{single}} \tag{S50}$$

where n is a conversion factor to convert concentrations to number of molecules (see Table A), $conc_{myo}$ is the concentration of active myosin II motors, and $F_{myo_{single}}$ is the force produced by a single myosin II motor. $conc_{myo}$ was adjusted such that the lowest value of velocity achieved for the baseline model was within 10% of that observed in experiments [22]. As a continuous ODE framework is used, we consider the same actin retrograde velocity for all clutches.

Force quantification The force on a clutch depends on its stiffness which depends on the number of constituent IAPCs and the number of vinculin molecules in each IAPC (see 'Stiffness of an integrin-adaptor protein complex'). As clutches are connected in series to the substrate spring, when actin filaments pull the system of springs by unit distance, the extension is shared between the clutch and the substrate (Fig 1C). The fractional extension a_{clutch} (for calculation, see section 'Fractional extension of clutches') experienced by the clutch when the entire system experiences unit extension, depends on the ratio of the stiffness of the clutch to the stiffness of the entire substrate–clutch system.

The total force on the network of actin filaments depends on the number of AB clutches of each type and the extensions of the respective clutches. Since we use a continuum approach to account for the abundance of each species, the concentrations need to be discretised. The need to discretise arises as the model treats the different clutches and force-exerting species as springs of certain stiffnesses. Thus, the force generated depends on the number of springs of a certain stiffness that experience an extension.

Concentrations are discretised by assuming a volume of 1 μm^3 which is approximately the volume of large focal adhesions and their immediate surroundings [23]. The concentrations were multiplied by the volume and the Avogadro number, N_A, accounting for the different units to obtain a discrete number of clutches. As mentioned earlier, all members of a certain species experience the same extension and force. The force exerted by one clutch of a particular species is

$$F_{clutch} = k_{clutch} \cdot (a_{clutch} \cdot v_{retro} \cdot dt) \tag{S51}$$

Where k_{clutch} is the stiffness of the clutch, a_{clutch} is the fractional extension of the clutch (derivation in section 'Fractional extension of clutches'), v_{retro} is the retrograde velocity in the current time step and dt is the duration of the time step. Then, the total force exerted by all clutches of this type is

$$F_{total_{clutch}} = m_{clutch} \cdot F_{clutch} \tag{S52}$$

where m is the number of clutches of a particular type i.e., the discretised concentration.

Therefore, the total force exerted by all AB clutches in the system is given by:

$$F_{total} = \sum_{clutch} m_{clutch} \cdot F_{clutch} \tag{S53}$$

Substrate rigidity range A range of substrate rigidities based on previous computational studies, between 0.1 pN/nm and 100 pN/nm, was used for simulations in this model [22]. For all investigations except parameter sensitivity analysis, four stiffnesses, namely k_{sub} =[0.1,1,10,100] pN/nm were used. For parameter sensitivity

analysis and to plot Fig 4 and Fig 5, 519 values of k_{sub} spaced approximately logarithmically between 0.1 pN/nm and 1000 pN/nm were used to obtain a more continuous distribution of stiffnesses.

To compare our simulated results with experimental results, substrate stiffness when expressed in terms of a Young's modulus was converted to a Hookean spring constant according to

$$k_{substrate} = \frac{4\pi r}{9} E \tag{S54}$$

where E is the Young's modulus, and r is the radius of a circular adhesion site (r=550 nm) as described in [24] and [25].

Signal-dependent rate modification (SDRM) Numerous nascent adhesions (NAs) form at the moving edge but only a few remain after a few minutes [11, 26]. The assembly of NAs correlates with cell protrusion speed and actin polymerization is necessary for the nucleation of NAs [11, 27]. Actin network branching reduces away from the cell edge [28, 29]. Since actin is depolymerized more rapidly away from the cell membrane [29] not all the NAs that were formed can be supported without an extensive actin filament network, and hence disassemble. Many signalling molecules such as focal adhesion kinase (FAK), Src and ERK kinases are known to influence adhesion disassembly. FAK phosphorylated at Tyr-397 was found to be positively correlated to adhesion disassembly rates [30]. FAK is also inhibited closer to the leading edge of the cell due to its interactions with Arp2/3, a protein involved in actin branching [31]. Actin branching reduces away from the leading edge [28, 29], implying that FAK is less inhibited in regions further from the leading edge. Since NA disassembly occurs as they move away from the leading edge, FAK may be implicated in NA disassembly. Additionally, calpain is known to be involved in regulating adhesion disassembly through proteolysis of talin [32], and its inhibition leads to disruption of adhesions containing zyxin [33].

Here, we model a hypothetical signal molecule which is essential for new NA formation (Rx1, Rx2, Rx3, Table B) and low-order AUB clutches (S1, C1) to bind actin (for maturation) (Rx4, Rx9, Table B). We impose that a minimum concentration, $signal_{thresh}$, of the signal molecule is required for these reactions to occur. The concentration $[signal]$ of this molecule is initially high and decreases according to eq. 17 (main text). Thus, the rate of decay of $[signal]$ determines the amount of time available before NA disassembly starts in which adhesion maturation can occur. The model behaviour does not change by assuming the opposite i.e., $[signal]$ increases over time and there is an upper limit for its concentration beyond which actin binding does not occur (Fig C.B).

The modifications to the rates were made by multiplying the baseline rate constant by a signal-dependent rate modification (SDRM) factor given by the function:

$$sig_{dep} = \begin{cases} 1 & [signal] > [signal_{thresh}] \\ \left(\frac{1}{[signal_{thresh}]}\right) \cdot [signal] & [signal] \leq [signal_{thresh}] \end{cases} \tag{S55}$$

This results in sig_{dep} having a value of 1 before $signal_{thresh}$ is crossed but would then decrease at the same rate as $[signal]$ (Fig C.A).

Thus, if the $[signal]$ falls below $signal_{thresh}$, the rates of the following reactions were modified as described:

$$\text{Pre-complex formation (Rx1)}: k_{1f} = k_{1f_{base}} \cdot sig_{dep} \tag{S56}$$
$$\text{S1 formation from Pcomp (Rx2)}: k_{2f} = k_{2f_{base}} \cdot sig_{dep} \tag{S57}$$
$$\text{C1 formation from S1 (Rx3)}: k_{3f} = k_{3f_{base}} \cdot sig_{dep} \tag{S58}$$
$$\text{Actin-binding rate (Rx4, Rx9)}: k_{4f} = k_{9f} = k_{act} \cdot sig_{dep} \tag{S59}$$

where k_{act} is the baseline actin-binding rate.

The breakdown rates of mid-order clutches (S2, C2) to low-order clutches (S1, C1) also decrease after [*signal*] falls below the threshold to reflect the fact that the adhesions that are chosen to be matured are not broken down, and may progress to form FAs. As FAs are much larger in size compared to NAs, it implies that the large clusters (C3, C2) are less likely to break down to smaller clusters (S3, S2) if they are undergoing maturation to FAs. Thus, the rates of breakdown of high and mid-order clusts to seeds (C3 to S3, C2 to S2) were also made [*signal*]-dependent. These rates were modified in the following way:

$$\text{S2, C2 to S1, C1 breakdown rate (Rx18, Rx20)}: k_{18f} = k_{20f} = tal_{rf} \cdot sig_{dep} \tag{S60}$$
$$\text{C3 breakdown to S3 (Rx21)}: k_{21f} = k_{21f_{base}} \cdot sig_{dep} \tag{S61}$$
$$\text{C2 breakdown to S2 (Rx22)}: k_{22f} = k_{22f_{base}} \cdot sig_{dep} \tag{S62}$$

where tal_{rf} is the baseline talin-refolding rate.

Once the signal concentration goes below $signal_{thresh}$, mechanisms to disassemble the initially formed clusters must become more active. Without an increase in activity, integrins will accumulate in low order AUB clutches (S1, C1) since the rate of breakdown of S1 to Pcomp is a very small value at baseline. To ensure these species are broken down once $signal_{thresh}$ is crossed, the reverse rates of Pcomp formation (Rx1), seed formation (Rx2), and seed dimerization (Rx3) need to be increased. These rates are modified in the following way:

$$\text{C1 breakdown (Rx3)}: k_{3r} = \begin{cases} k_{3r_{base}} & [signal] > [signal_{thresh}] \\ k_{dis} \cdot (1 - sig_{dep}) & [signal] \leq [signal_{thresh}] \end{cases} \tag{S63}$$

$$\text{S1 breakdown (Rx2)}: k_{2r} = \begin{cases} k_{2r_{base}} & [signal] > [signal_{thresh}] \\ k_{dis} \cdot (1 - sig_{dep}) & [signal] \leq [signal_{thresh}] \end{cases} \tag{S64}$$

$$\text{Pcomp breakdown (Rx1)}: k_{1r} = \begin{cases} k_{1r_{base}} & [signal] > [signal_{thresh}] \\ 2 \cdot k_{1r_{base}} \cdot (1 - sig_{dep}) & [signal] \leq [signal_{thresh}] \end{cases} \tag{S65}$$

The value of k_{dis} was obtained by multiplying the experimentally measured disassembly rate [11] by a factor such that the simulations closely matched the experimentally determined NA assembly-disassembly curves [11] (Fig 4, main text).

Slip and catch bonds The talin–actin bond is a slip bond whose stability decreases with increasing force [3], implying a monotonically increasing unbinding rate with force. Here, we assume the unbinding rate increases exponentially with force according to the Bell model [34]. The stability of catch bonds increases with the application of force [35]. With integrins, both catch and slip bond characteristics were observed in different force regimes, leading to the concept of a catch-slip bond. Particularly, the catch-slip behaviour was observed for $\alpha_5\beta_1$, $\alpha_4\beta_1$ and $\alpha_L\beta_2$ integrins under different force

regimes [36–38]. In a catch-slip bond, the lifetime of the bond increases up to a force threshold beyond which the lifetime starts to decrease. A fully connected force chain (ligand–integrin–adaptor protein–actin filament) can be broken at two points, namely at the talin–actin slip bond or the integrin–ligand catch-slip bond. In this model, integrins are assumed to be ligand-bound when they are actin-bound. Thus, an actin-bound clutch is considered to become actin-unbound when either the integrin-ligand bond or the talin-actin bond is broken. The effective rate of actin-unbinding of a particular clutch depends on the force experienced by individual complexes in the clutch and is given by a sum of the catch-slip and slip bond rates:

$$\{k_{4\mathrm{r}}, k_{5\mathrm{r}}, k_{6\mathrm{r}}, k_{9\mathrm{r}}, k_{10\mathrm{r}}, k_{11\mathrm{r}}\} = A \cdot e^{-b \cdot F_{clutch}} + C \cdot e^{d \cdot F_{clutch}} + k_{slip_{UL}} \cdot e^{\frac{F_{clutch}}{F_{th_i}}} \qquad \text{(S66)}$$

where F_{clutch} is the force on an individual complex in the clutch, F_{th_i} is the talin–actin slip bond rupture threshold ($i \in \{1, 2, 3\}$ (Table A), $k_{slip_{UL}}$ is the unloaded dissociation rate of talin from actin. The first and second terms define the integrin-ligand catch-slip bond - $A \cdot e^{-b \cdot F_{clutch}}$ captures the decrease in unbinding rate characteristic of a catch bond and $C \cdot e^{d \cdot F_{clutch}}$ captures the increase in unbinding rate characteristic of a slip bond, b and d are parameters that control the force-dependency of the unbinding rate.

Since vinculin also binds actin, reinforcement increases the force-carrying capacity of a clutch. The vinculin–actin bond is a catch-slip bond that is maximally stable at 8 pN of force [2]. The talin–actin slip bond is the weaker link of these two, with a rupture force of 2 pN [3]. Therefore, for any increase in force up to 8 pN, the vinculin–actin unbinding rate reduces, effectively maintaining the actin-bound state of a clutch for longer. To maintain simplicity, instead of explicitly modelling the vinculin–actin catch bond, reinforcement was modelled as an increase in the force thresholds for the talin-actin slip bond. The force thresholds were increased by 0.5 pN for each reinforcement event, effectively increasing the force thresholds for mid (S2a, C2a) and high (S3a, C3a) order AB clutches, F_{th_2} and F_{th_3}, to 2.5 and 3 pN respectively (Table A). Once the force on AB clutches reaches the corresponding force threshold, they are assumed to immediately unbind from actin to give their AUB counterparts, resulting in the concentration of AB clutches going to 0, and that of AUB clutches increasing by the same amount.

Time-dependent rate modification (TDRM) Although integrins cluster on substrates of all stiffnesses, the traction force generated and consequently the maturation of adhesions depends on the substrate stiffness [22, 24, 39]. Once unbound, the talin in the clutch returns to its folded state and the unfolding process needs to start again. While this can result in an equilibrium being established with a large number of simultaneously bound clutches, the force on each clutch remains low and only increases slowly. Therefore, even though the number of bound clutches (or concentration) is high, the average force per clutch (total force divided by the number of bound clutches) remains low as the clutches are likely to unbind spontaneously before reaching their maximum force-carrying capacity.

The model in this study is a continuous model that aims to capture the overall 'average' behaviour of all clutches. As such, all clutches of a particular kind are said to experience the same force and this increases with every time-step until the respective thresholds are reached. Thus, the total force exerted by a certain clutch type is the product of the concentration and the force on one such clutch. This makes it challenging to account for the reduction in the total force that is caused by spontaneous clutch unbinding events as it is impossible to keep track of force on individual clutches. To account for this we introduce a method of time dependent rate modification

(TDRM) to capture the (individual, discrete) spontaneous clutch unbinding events in a continuous framework. Since the total force exerted by a particular family of clutches is the product of the concentration of the clutch and the force on one such clutch, by introducing a time-dependent increase in the unbinding rate, it is possible to ensure that the total force does not monotonically increase.

More specifically, as derived and described by Novikova et al. [40], the probability p_{closed} that a bond that is closed at time t remains closed at time $t + \Delta t$ reduces exponentially and is approximately equal to:

$$p_{closed}\,(t{+}\Delta t)\ \sim e^{-k_{LT}\cdot\ \Delta t} \tag{S67}$$

where $\frac{1}{k_{LT}}$ is the expectation value of the lifetime [40] (this theoretical formulation is only used to support our formulation of TDRM, and we do not use the expectation value k_{LT} in our model). Thus, the probability of bond-rupture increases with time spent in the bonded state, Δt, asymptotically reaching the value of 1. Hypothetically, as the probability of bond-rupture p_{open} approaches 1 (p_{closed} approaches 0), the bond-rupture rate approaches infinity causing all bound clutches to unbind. Thus, the bond-rupture rate grows exponentially with decreasing p_{closed}. In this model, the time step dt is fixed (Table A). Because we use Euler's forward integration method, the concentrations at time $t + \Delta t$ are given by $rate \cdot dt$. Thus allowing an unlimited, exponential growth in reaction rates can lead to numerical errors such as negative concentrations. To limit the unbinding rates and prevent numerical integration errors but still preserve the monotonic increase, we restrict the increase in unbinding rates to a linear regime. This way, the maximum value of $rate \cdot dt$ for the time scales of this model is not too large, and numerical integration errors are avoided. With this approach, the model still qualitatively captures the effects of an increasing unbinding rate, but the effect will not be as pronounced as it would be if an exponential function was used. Nevertheless, even with this restriction to linear increases for TDRM, our results highlight the importance of such a mechanism. In future work, adaptive time stepping can be used with exponential increases to more accurately represent the underlying biological processes.

Thus, TDRM was incorporated by modifying the rate constants for actin-unbinding reactions (k_{4r}, k_{5r}, k_{6r}, k_{9r}, $k_{10\mathrm{r}}$, k_{11r}) by multiplying the unloaded dissociation rate of the slip bond, $k_{slip_{UL}}$ with a TDRM factor given by:

$$k_{TDRMFactor} = 1 + k_{sens} \cdot t_{clutch} \cdot dt \tag{S68}$$

where k_{sens} determines the sensitivity to the time t_{clutch} that AB seeds or AB clusts spend in the force-loading phase. t_{clutch} increases by 1 in each time step until the force F_{clutch} on each complex in the clutch equals $F_{th_{clutch}}$, the threshold force, when it is reset to 0. The value of k_{sens} can be taken to represent the reciprocal of the lifetime of the bond just as k_{LT} in eq. S67. The lifetime of the talin-actin bond under physiological conditions is between 10–100 seconds [41]. Here, a baseline value of k_{sens}=0.05 is used (implying a lifetime of 20 seconds), but it is important to note that the qualitative trends in the model outcomes remained the same for values of $k_{sens} \geq 0.02$ (lifetime ≤ 50 seconds).

Since the chain of links between the ECM and the actin cytoskeleton can break at the integrin–ECM or the talin–actin linkages, an increase in either the integrin–ECM catch-bond rupture or talin–actin slip bond rupture in essence has the same effect on the results of the model – both lead to an incomplete chain and subsequent loss of force transmission. As TDRM was devised to qualitatively capture bond-rupture dynamics, we limited the effect of TDRM to the unloaded slip bond dissociation rate $k_{slip_{UL}}$. However, increasing both the unloaded slip and catch-bond dissociation rates ($k_{slip_{UL}}$, A and C in Eq S66) also results in the same trends but with a larger difference between

concentrations of species on soft and stiff substrates, and consequently the maturation fraction (Fig M)

Curve-fitting for parameter values The values of parameters of the integrin–ligand catch bond and the rate of talin unfolding due to force were determined by fitting double and single exponential equations respectively to experimental data [16, 37] using the in-built MATLAB function 'fminsearch'. Specifically, for the catch bond, force-bond lifetime data of the Mn^{2+} activated $\alpha_5\beta_1$- Fn (fibronectin) from Kong et al. (2009) [37] was obtained by digitizing Figure 3C in the publication. For the rate of talin unfolding, data from experiments involving stretching of full-length talin by Rio et al. (2009) [16] was used (Fig 4F in the main text).

The stiffness of talin used in this study (0.1 pN/nm) was obtained by calculating the gradient of force-extension curves in the linear regime at low extensions of full length talin rod as reported by Yao et. al (2016) [15].

Mass conservation Mass conservation was checked by calculating the sum of concentrations of integrins and talin across all integrin and talin containing species in the model. The equations for mass conservation are below:

$$\begin{aligned}
\text{Integrins}: [int] + [Pcomp] + 25 \cdot ([S1] + [S2] + [S3] + [S1a] + [S2a] + [S3a]) \quad &\text{(S69)}\\
+50 \cdot ([C1] + [C2] + [C3] + [C1a] + [C2a] + [C3a])&\\
\text{Talin}: [tal] + [Pcomp] + 25 \cdot ([S1] + [S2] + [S3] + [S1a] + [S2a] + [S3a]) \quad &\text{(S70)}\\
+50 \cdot ([C1] + [C2] + [C3] + [C1a] + [C2a] + [C3a])&\\
\text{Vinculin}: [vinc] + [Pcomp] + 25 \cdot ([S1] + [C1] + [S1a] + [C1a]) \quad &\text{(S71)}\\
+50 \cdot ([S2] + [S2a] + [C2] + [C2a])&\\
+75 \cdot ([S3] + [S3a] + [C3] + [C3a])&
\end{aligned}$$

Fitting a line through force peaks For Fig K, to quantify the peak force exerted by each species and the total force exerted by all species over time, the peaks of the force-time data were identified using a built-in MATLAB function 'findpeaks'. A smoothing spline was then fit through the identified peaks using the 'fit' function in MATLAB.

Text B. Results

Mass conservation Testing for mass conservation showed that the total concentration of integrins and talin during the simulation remained at the initial concentrations of 1 µM (Fig B). This was also true for vinculin when the simulation was run with a limited amount of vinculin (not shown). This shows that there are no runaway variables or leaks in the system of differential equations used.

Signal decay (or growth) rate matches nascent adhesion assembly phase Fig C.A shows the concentration of [*signal*] over time. The rate of decay of the signal molecule was adjusted such that it crosses the concentration threshold $signal_{thresh}$ (of 0.1 μM) after around 58 seconds. t_{sig} is the time at which this threshold is crossed. NA formation reactions are allowed to undergo for this duration as it is the length of the experimentally determined NA assembly phase [11].

The behaviour of the model does not change if the converse is assumed – i.e. the [*signal*] grows with time and above a certain concentration of [*signal*] (here the

threshold is set to 0.9 μM) the signal-dependent reactions start to slow down. The growth rates can be adjusted to result in the same time dynamics (Fig C.B).

Limited vinculin results As the simulations by default assume a constant concentration of vinculin, to investigate whether the optimal substrate stiffness is obtained even in limited vinculin environments, the initial concentration of vinculin was set to 1 uM, equal to that of integrin and talin, and was not replenished during the simulation. The rest of the parameters were maintained at baseline values. The general trends for concentrations of mid and high order clutches remained the same with limited vinculin concentration (Fig E). A key difference was that the concentration of high-order clutches was lower by almost an order of magnitude (Fig E(iii) vs Fig G.A(iii)) and the concentration of low-order clutches was nearly twice as high compared to constant vinculin conditions (Fig E(i) vs Fig G.A(i)). This coupled decrease and increase in concentrations show that the seed and clust ([S1], [C1], [S1a], [C1a]) formation occurred to the same extent, but maturation was lower when vinculin was limited. While the maturation fraction varied biphasically with substrate stiffness when the vinculin concentration was kept constant (Fig G.A(iv)), there was a monotonic decrease when the vinculin concentration was limited (Fig E.(iv)). Increasing k_{sens} increased this difference (not shown).

Investigating the biphasic relation between traction force and substrate stiffness To ensure that the biphasic relationship between force and substrate stiffness indeed arises from a combination of low force and low concentrations of clutches, we can look at the total force exerted by each clutch on substrates of different stiffnesses. The total force exerted by a species, which we term 'force-concentration' for simplicity, is the product of the discretised concentration of the species and the force on one clutch of the species (S57). To capture the general trend, a curve can be fitted on the peaks of the force-concentration plots. Fig K shows these fitted curves for each species on four substrate stiffnesses. Notably, the force-concentration of C3a and S3a are the highest on all stiffnesses as they are the most reinforced clutches and have the highest force-carrying capacities. The force-concentration of S3a is higher than that of C3a initially even though the force-capacity of C3a is twice that of S3a (as C3a is a dimer of S3a). But there is a gradual reduction of this difference over time due to continued dimerization of S3a into C3a.

Sensitivity analysis results Fig I and Fig J show the results of the sensitivity analysis conducted on 21 different parameters on the two outcomes detailed in the main text. Of these, 7 parameters were chosen to be shown in Fig 5 (main text), either because the outcomes were highly sensitive to these parameters or because they were of particular interest although they had a relatively low influence.

Differences in trends of concentrations of [S1a] and [C1a] The model predicts highest [S1a] and [S3a] to be on the 100 pN/nm and 1 pN/nm substrates respectively (Fig 3B), whereas [C1a] and [C3a] are always higher on 1 pN/nm. Given that in the absence of maturation reactions, [S1] and [C1] are rigidity-independent, this highlights the difference in reinforcement rates between stiffnesses. The rate of consumption of [S1a] is lowest on the 100 pN/nm substrate as the clutches reach the talin-actin slip bond threshold rapidly, leaving little time for vinculin-binding reinforcement reactions, and hence clutches accumulate in this state. This explains the lower [S2a] and [S3a] on stiff substrates. However, the trend of [C1a] on different stiffnesses is explained to be a result of mass action kinetics as follows. When the dimerization of S3a to C3a (Rx16) and the breakdown of C3 to S3 (Rx21) are disabled, the trends for [S1a] and [C1a] on

different stiffnesses are the same (Fig N). When these reactions are enabled, there is an increase in [C3a] on all stiffnesses. The highest change (fold-change of ~ 6) is on $k_{sub} =$ 1 pN/nm (Fig 3B), while for other stiffnesses the fold-change is roughly between 2 and 3. This considerably larger increase on $k_{sub} = 1$ pN/nm results, due to mass action kinetics, in a higher [C2a] and [C1a] on $k_{sub} = 1$ pN/nm compared to other stiffnesses. While this change in trends of [C1a] and [S1a] on different stiffnesses is attributable to the choice of kinetics in the model, the absolute values of the concentrations and the differences therein are two orders of magnitude smaller than the concentrations of the other species. As such, although the mature and nascent stages of adhesions are mathematically coupled due to the mass-action kinetic framework we use, the effect thereof appears to be minor and limited. Note that in reality these stages and transitions between them are likely to be tightly regulated by other factors to be identified experimentally and currently not included in the model.

Table A. Parameter values used for the baseline model

Parameter	Definition	Value	Ref.	Remark	Ref. type
$k_{1f_{base}}$	Pre-complex formation	0.12 s^{-1}	[42, 43]		*Experimental*
k_{1rbase}	Pre-complex dissociation	0.095 s^{-1}	[42]		*Experimental*
$k_{2f_{base}}$	S1 formation from Pcomp	0.021 s^{-1}	[11]		*Experimental*
$k_{2r_{base}}$	S1 dissociation to Pcomp	0.0001 s^{-1}		*Estimate*	
$k_{3f_{base}}$	C1 formation from S1	0.021 s^{-1}	[11]		*Experimental*
$k_{3r_{base}}$	C1 dissociation to S1	0.0001 s^{-1}		*Estimate*	
k_{7r}	Vinculin dissociation from AB *seed*	0.0001 s^{-1}		*Estimate*	
k_{8r}	Vinculin dissociation from AB *seed*	0.0001 s^{-1}		*Estimate*	
k_{12r}	Vinculin dissociation from AB *clust*	0.0001 s^{-1}		*Estimate*	
k_{13r}	Vinculin dissociation from AB *clust*	0.0001 s^{-1}		*Estimate*	
k_{14f}	S1a dimerization	1 s^{-1}	[44]		*Computational*
k_{14r}	C1a dissociation to S1a	0.001 s^{-1}		*Estimate*	
k_{15f}	S2a dimerization	1 s^{-1}	[44]		*Computational*
k_{15r}	C2a dissociation to S2a	0.001 s^{-1}		*Estimate*	
k_{16f}	S3a dimerization	1 s^{-1}	[44]		*Computational*
k_{16r}	C3a dissociation to S3a	0.001 s^{-1}		*Estimate*	
$k_{21f_{base}}$	C3 dissociation to S3	0.005 s^{-1}		*Estimate*	
$k_{22f_{base}}$	C2 dissociation to S2	0.008 s^{-1}		*Estimate*	
$k_{23_{KM}}$	Michaelis-Menten constant for [*signal*] decay	2.13 μM		*Estimate*	
$k_{23_{vmax}}$	Maximum velocity for [*signal*] decay reaction	0.1 $\mu M s^{-1}$		*Estimate*	
$k_{unfold_{UL}}$	Rate of unloaded talin unfolding	1.54 s^{-1}	[16]		*Experimental*
$k_{off_{UL}}$	Unloaded talin-actin unbinding rate	0.35 s^{-1}	[22]	*Estimate based on literature*	*Computational*
k_{uf}	Talin-unfolding exponent factor	0.05	[16]		*Experimental*
A	Catch bond parameter	2.52 s^{-1}	[37]		*Experimental*

b	Catch bond parameter	-0.107 s^{-1}	[37]		*Experimental*
C	Catch bond parameter	0.000123 s^{-1}	[37]		*Experimental*
d	Catch bond parameter	0.1897 s^{-1}	[37]		*Experimental*
F_{th1}	Force threshold for talin-actin slip bond for low order clutch	2 pN	[3]		*Experimental*
F_{th2}	Force threshold for talin-actin slip bond for mid order clutch	2.5 pN	[3]	*The increase is estimated to account for vinculin-actin bond*	*Experimental*
F_{th3}	Force threshold for talin-actin slip bond for high order clutch	3 pN	[3]	*The increase is estimated to account for vinculin-actin bond*	*Experimental*
F_{vb1}	Force threshold for 1st vinculin binding event	5 pN	[15]		*Experimental*
F_{vb2}	Force threshold for 2nd vinculin binding event	12 pN	[16]		*Experimental*
$F_{myo_{single}}$	Force exerted by a single myosin motor	2 pN	[20]		*Experimental*
v_u	Unloaded actin retrograde velocity	110 nm/s	[22, 31]		*Computational, Experimental*
Local volume	Volume of focal adhesions and their immediate surroundings	1 um^3	[23]	*Estimate*	*Experimental*
k_{tal}	Stiffness of talin molecule	0.1 pN/nm	[15]	*Estimate from force-extension curves*	*Experimental*
k_{vinc}	Stiffness of vinculin molecule	0.25 pN/nm	[2]		*Experimental*

tal_{rf}	Talin refolding rate	$1\ s^{-1}$	[15]	*Estimate based on experimental data (Fig 5 e)*	*Experimental*
$tal_{rf_{factor}}$	Talin refolding rate factor for 1st refolding event	0.5		*Estimate*	
k_{dis}	Disassembly rate of nascent adhesions	$0.02434\ s^{-1}$	[11]	*Adjusted – experimental rate was multiplied by 2*	*Experimental*
k_{sub}	Substrate stiffness	0.1-100 pN/nm		*Tested*	
k_{sens}	Time-dependency factor	0.05		*Tested*	
dt	Time step	0.005 s		*Used in this study*	
n	Conversion factor from concentration to molecules	Local volume*N_A			
$conc_{myo}$	Concentration of active myosin motors	$4\ \mu M$		*Adjusted*	
$signal_{thresh}$	Threshold concentration of $[signal]$	$0.1\ \mu M$		*Arbitrary*	
N_A	Avogadro's constant	$6.023 \cdot 10^{23}$			

Table B. Reactions in the model

Rx number	Reaction
Rx1	$k_{1f} \cdot [int] \cdot [tal] \cdot [vinc] - k_{1r} \cdot [Pcomp]$
Rx2	$k_{2f} \cdot [Pcomp]^2 - k_{2r} \cdot [S1]$
Rx3	$k_{3f} \cdot [S1]^2 - k_{3r} \cdot [C1]$
Rx4	$k_{4f} \cdot [S1] - k_{4r} \cdot [S1a]$
Rx5	$k_{5f} \cdot [S2] - k_{5r} \cdot [S2a]$
Rx6	$k_{6f} \cdot [S3] - k_{6r} \cdot [S3a]$
Rx7	$k_{7f} \cdot [S1a] \cdot [vinc]^2 - k_{7r} \cdot [S2a]$
Rx8	$k_{8f} \cdot [S2a] \cdot [vinc]^2 - k_{8r} \cdot [S3a]$
Rx9	$k_{9f} \cdot [C1] - k_{9r} \cdot [C1a]$
Rx10	$k_{10f} \cdot [C2] - k_{10r} \cdot [C2a]$
Rx11	$k_{11f} \cdot [C3] - k_{11r} \cdot [C3a]$
Rx12	$k_{12f} \cdot [C1a] \cdot [vinc]^2 - k_{12r} \cdot [C2a]$
Rx13	$k_{13f} \cdot [C2a] \cdot [vinc]^2 - k_{13r} \cdot [C3a]$
Rx14	$k_{14f} \cdot [S1a]^2 - k_{14r} \cdot [C1a]$
Rx15	$k_{15f} \cdot [S2a]^2 - k_{15r} \cdot [C2a]$
Rx16	$k_{16f} \cdot [S3a]^2 - k_{16r} \cdot [C3a]$
Rx17	$k_{17f} \cdot [S3]$
Rx18	$k_{18f} \cdot [S2]$
Rx19	$k_{19f} \cdot [C3]$
Rx20	$k_{20f} \cdot [C2]$
Rx21	$k_{21f} \cdot [C3]$
Rx22	$k_{22f} \cdot [C2]$
Rx23	$-\frac{k_{23_{vmax}} \cdot [signal]}{k_{23_{KM}} + [signal]}$

Fig A Hookean spring system of an individual Integrin-Adaptor Protein Complex (IAPC) used in this model. IAPCs bind in groups of 25 and 50 to form seeds and clusts respectively. A, B, C, and D together represent the talin rod, here modelled as 4 sub-domains. Springs E, G, and F represent vinculin molecules.

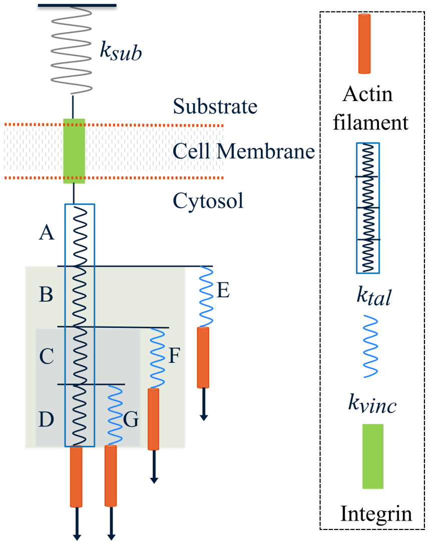

Fig B Mass conservation of integrins (A) and talin (B) is satisfied. The concentration remains at 1 μM for the entire duration of the simulation.

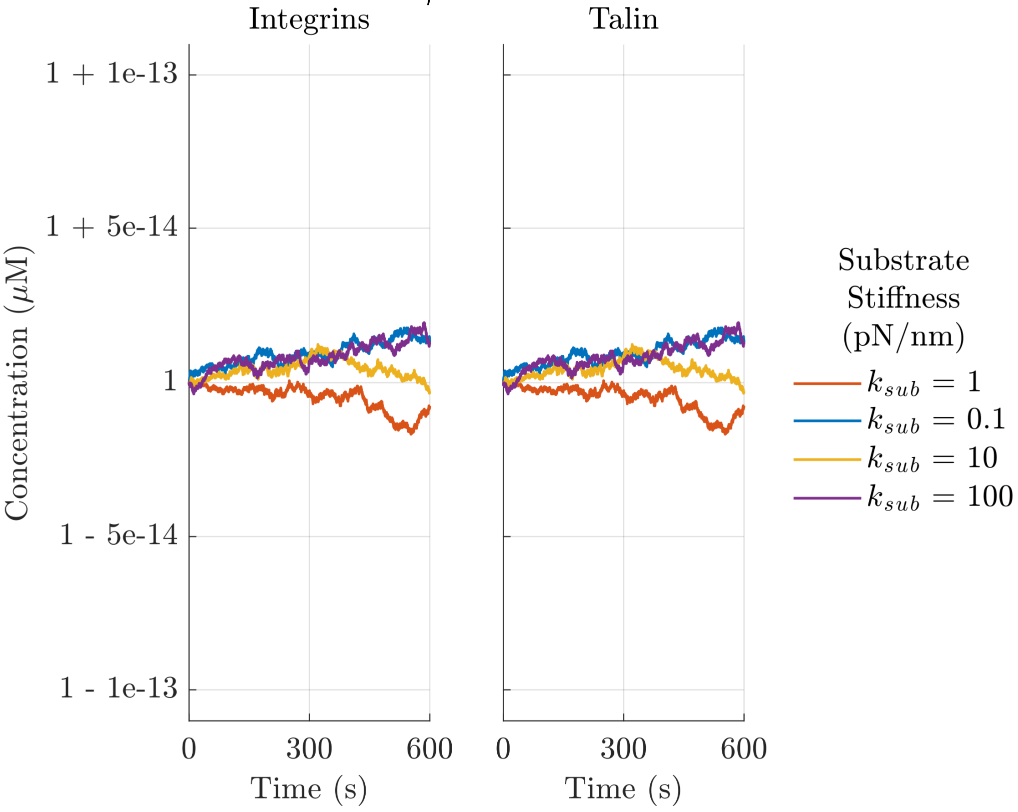

Fig C Concentration of [*signal*] (blue) over time. The yellow line represents the value of the SDRM factor, orange vertical line marks the time at which [*signal*] goes (A) below (or above (B)) $signal_{thresh}$ which is shown by the orange dashed line. t_{sig} is the time when [*signal*] crosses the threshold $signal_{thresh}$. A and B show signal decay and signal growth, respectively. The time t_{sig} and the SDRM factor are also identical in both.

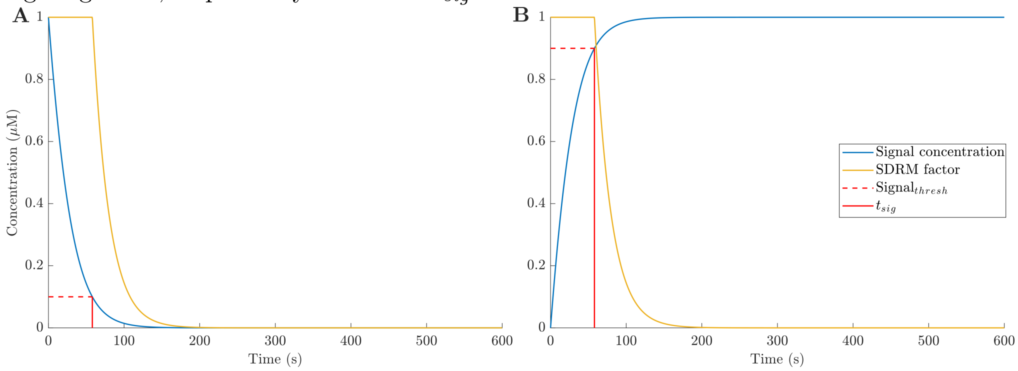

Fig D Concentrations of actin-bound species (i-vi) in the first 70 seconds of the simulation. The growth of mid and higher order seeds and clusts is most rapid on a substrate of moderate stiffness (k_{sub} =1 pN/nm).

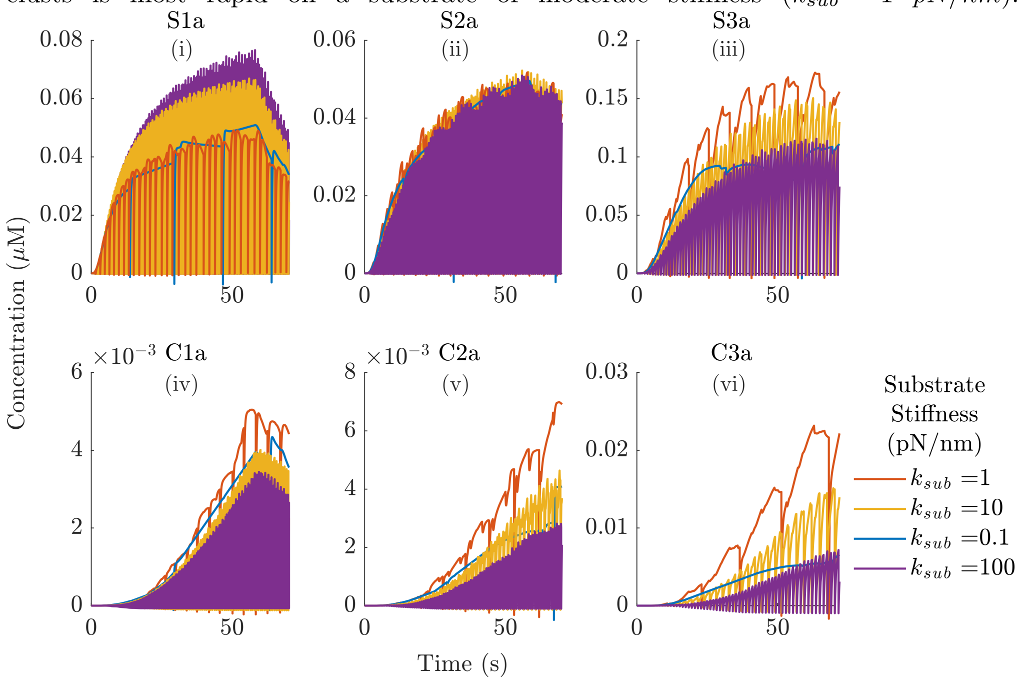

Fig E Limited vinculin test. Concentrations of integrins in (i) low-, (ii) mid-, and (iii) high-order species when vinculin concentration is limited to 1 μM. (iv) shows the maturation fraction (or the concentration of integrins in mature adhesions) increases monotonically with increasing stiffness.

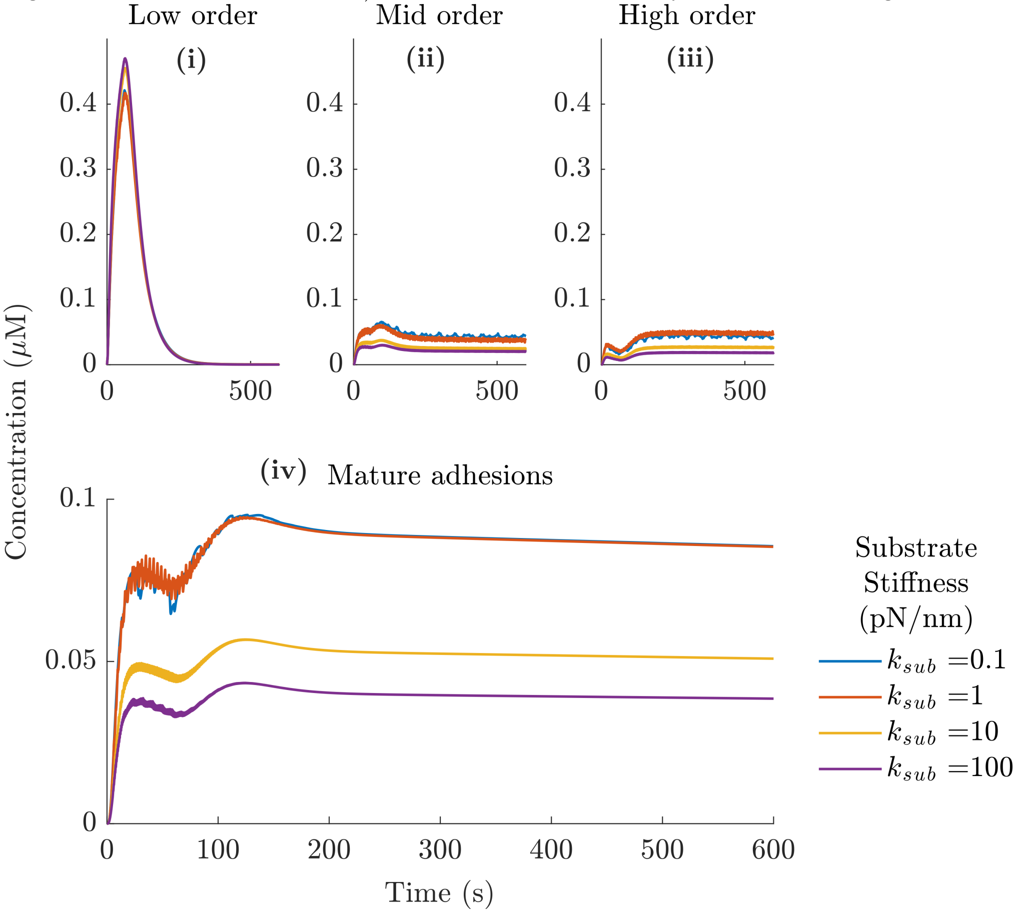

Fig F The effect of TDRM. Value of the actin-unbinding rates for the largest clusts C3a, k_{11r} (bottom) and the smallest seeds S1a, k_{4r} (top) over time for soft (A, C, E, G) and stiff (B, D, F, H) substrates for simulations with (A-D) and without (E-H) TDRM. The effect of TDRM is highly pronounced for $k_{sub} = 0.1\ pN/nm$ seen as a jump in the maximum value from $\sim 2.7\ s^{-1}$ *to* $\sim 6.8\ s^{-1}$ but negligible for $k_{sub} = 100\ pN/nm$ as the time period in actin-binding phase is very short.

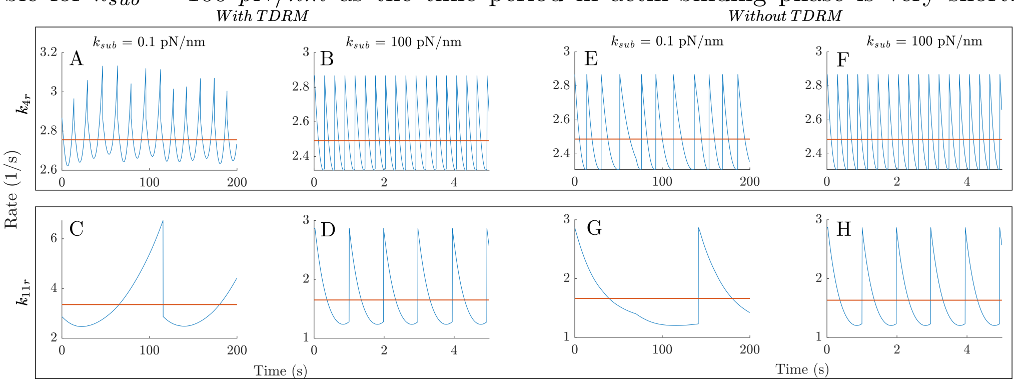

Fig G Concentrations of integrins in (i) low-, (ii) mid-, and (iii) high-order species in simulations with (A) and without (B) TDRM. (iv) shows the maturation fraction (or the concentration of integrins in mature adhesions). In A(iv), a biphasic trend is seen – the highest maturation fraction is on an optimal substrate stiffness of $k_{sub} = 1\ pN/nm$ (orange curves) whereas in B(iv), a monotonic decrease in maturation fraction is seen.

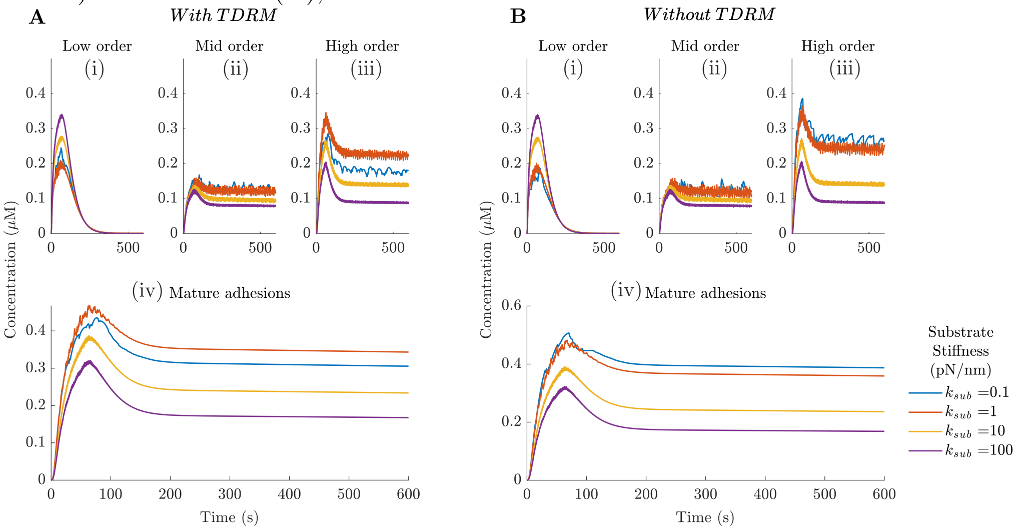

Fig H Concentrations of integrins in A(i) low-, A(ii) mid-, and A(iii) high-order species when the talin refolding factor $tal_{rf_{factor}}$ was set to 0.2/s. A(i–v) shows the maturation fraction (or the concentration of integrins in mature adhesions). B(i – vi) show the concentrations of actin-bound species under the same conditions.

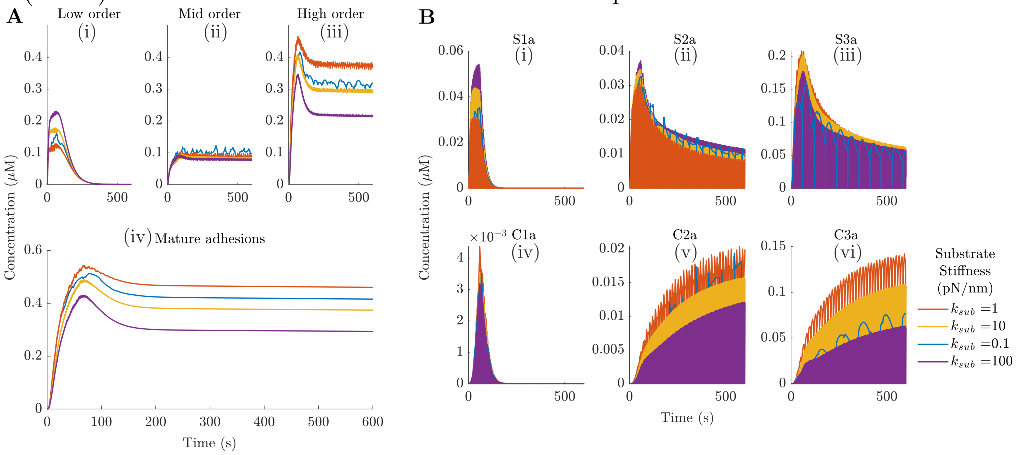

Fig I Sensitivity analysis for outcome 1: Maturation fraction. The 21 parameters are split into two panels A and B for ease of presentation. Sub-panels (a), (b), (c), and (d) represent the sensitivities of the outcome for changes of +20%, +10%, -10%, and -20% to the parameter values respectively. Refer to Table A for parameter descriptions.

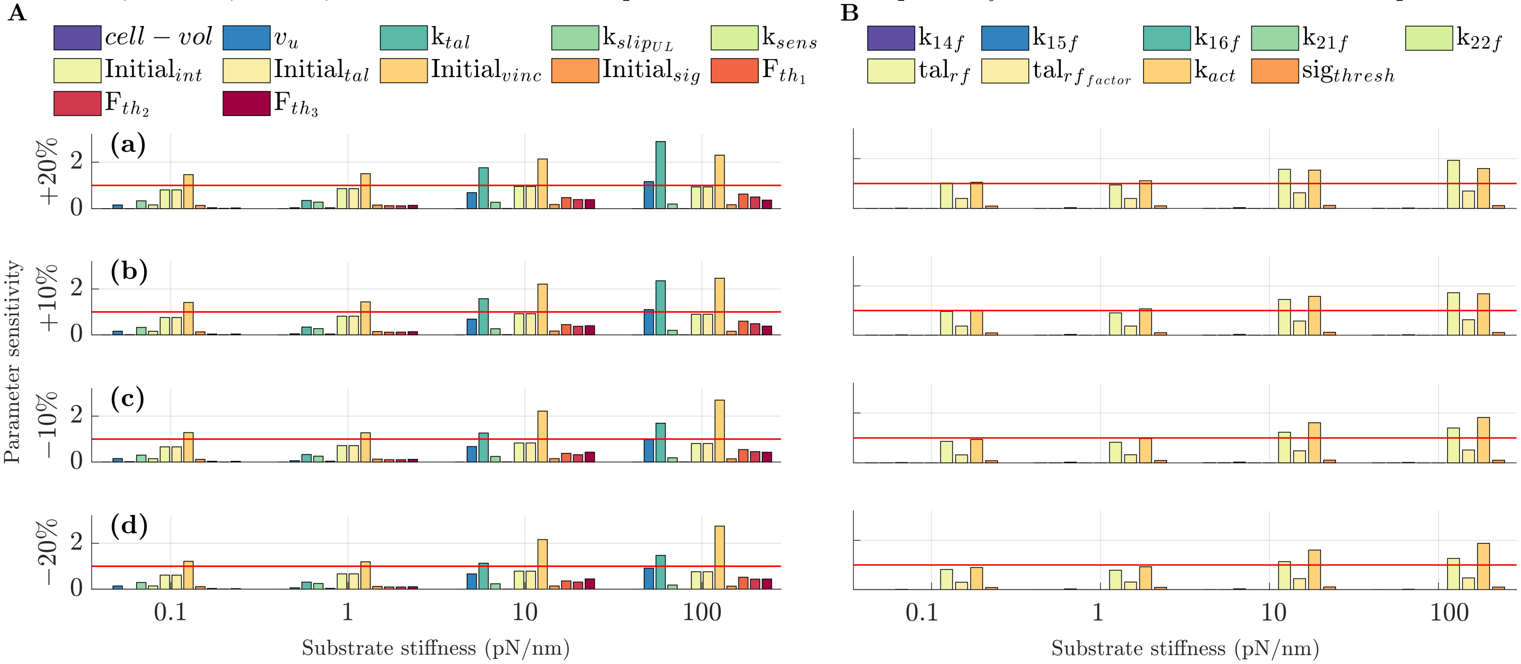

Fig J Sensitivity analysis for outcome 2: Optimum stiffness. The 21 parameters are split into two panels A and B for ease of presentation. Sub-panels (a), (b), (c), and (d) represent the sensitivities of the outcome for changes of +20%, +10%, -10%, and -20% to the parameter values respectively. Refer to Table A for parameter descriptions.

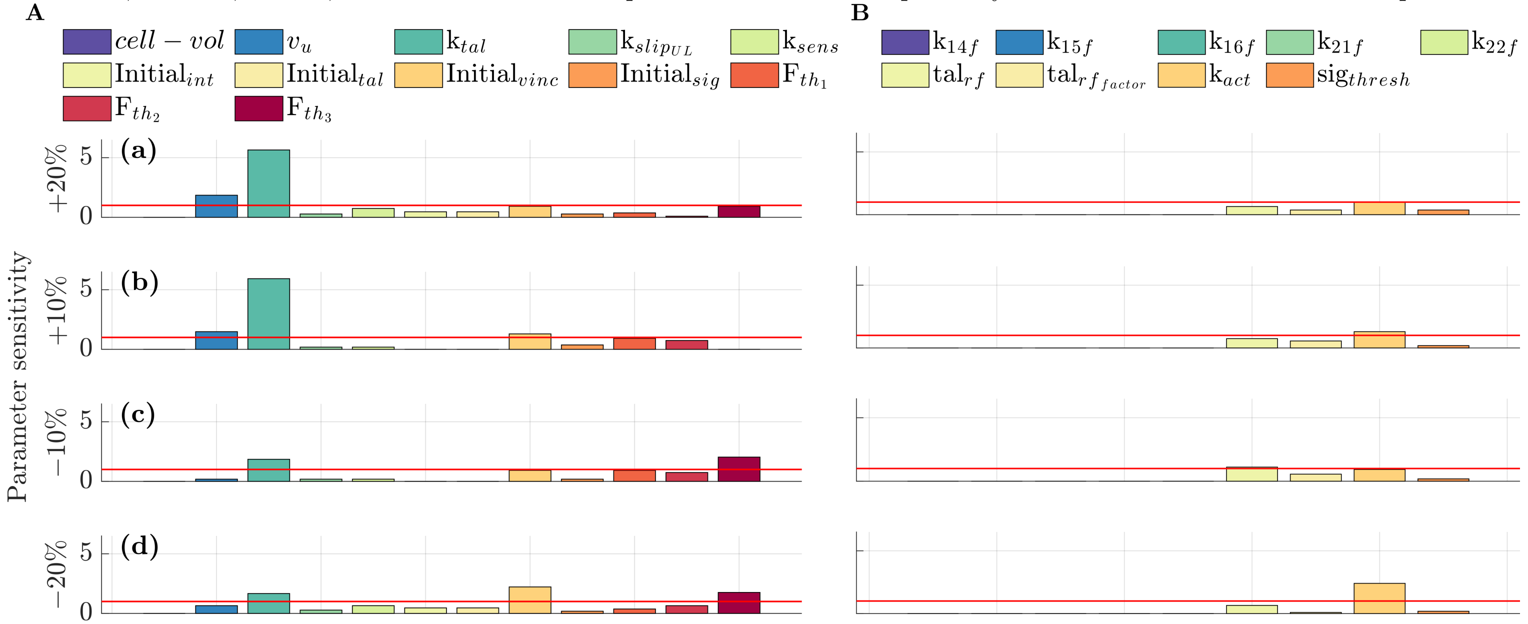

Fig K Curve fitting. Plots of the curve fitted over peaks of the force exerted by each species on substrates of varying stiffness (A-D). The total force exerted by all clutches on a particular substrate is shown by the black dotted line.

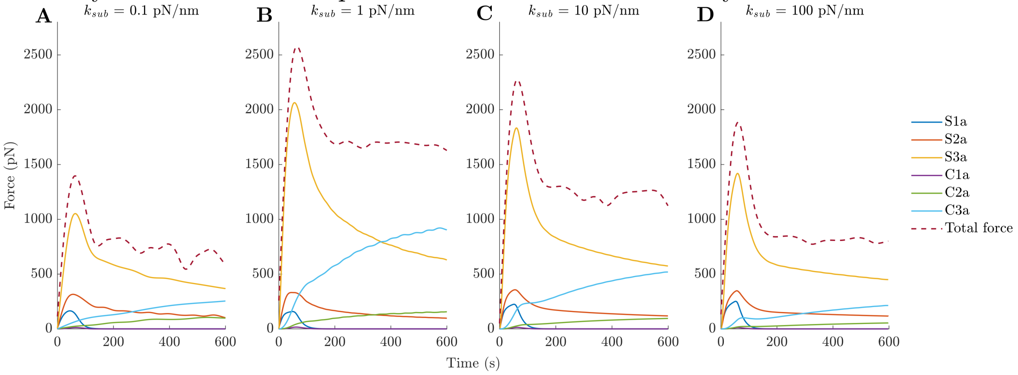

Fig L Concentrations of integrins in A(i) low-, A(ii) mid-, and A(iii) high-order species when the order of reinforcement reactions were set to 1. A(iv) shows the maturation fraction (or the concentration of integrins in mature adhesions). B(i – vi) show the concentrations of actin-bound species under the same conditions.

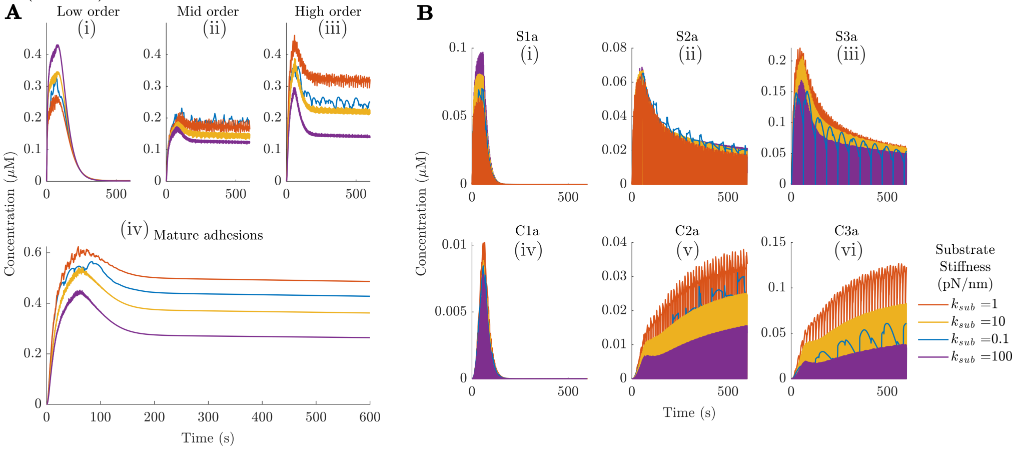

Fig M Concentrations of integrins in A(i) low-, A(ii) mid-, and A(iii) high-order species when both slip and catch bond dissociation rates are modified with TDRM. A(iv) shows the maturation fraction (integrins in mature adhesions). B(i–vi) show the concentrations of actin-bound species under the same conditions in Table A.

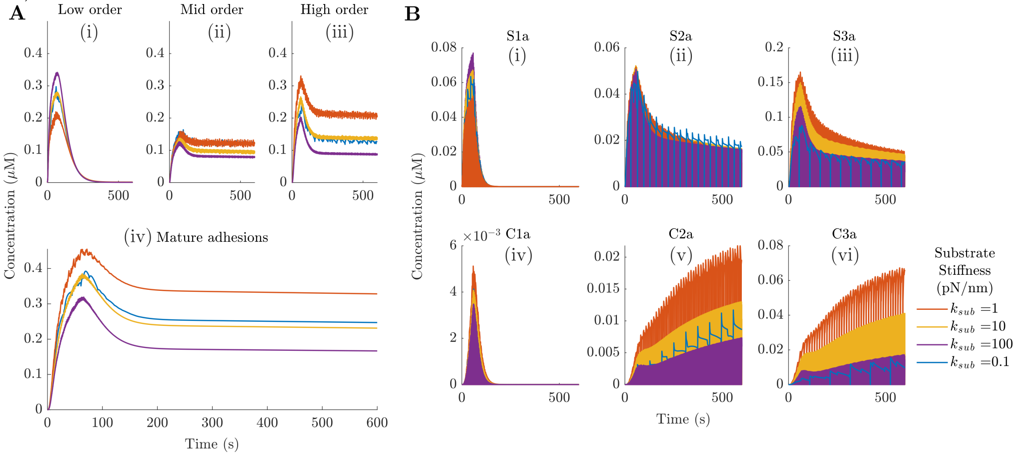

Fig N Concentrations over time of all the actin-bound species when the dimerisation of S3a (Rx 16) and breakdown of C3a to S3a (Rx 21) are disabled.

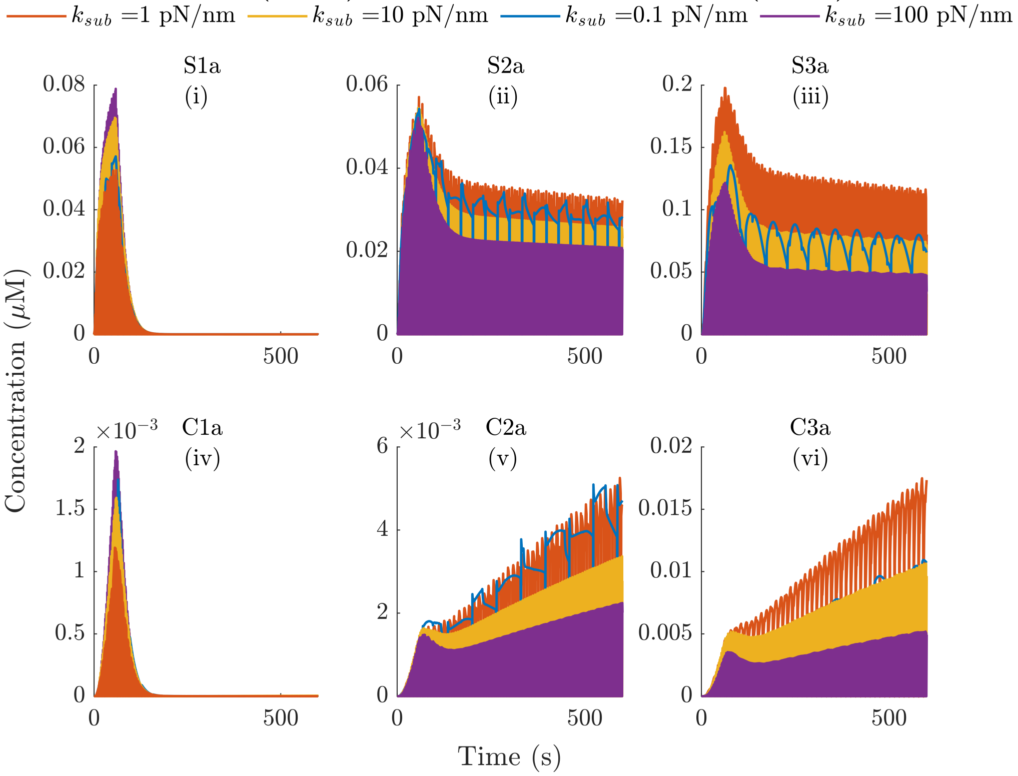

References

1. Han SJ, Azarova EV, Whitewood AJ, Bachir A, Guttierrez E, Groisman A, et al. Pre-complexation of talin and vinculin without tension is required for efficient nascent adhesion maturation. eLife. 2021;10. doi:10.7554/ELIFE.66151.

2. Huang DL, Bax NA, Buckley CD, Weis WI, Dunn AR. Vinculin forms a directionally asymmetric catch bond with F-actin. Science (New York, NY). 2017;357:703. doi:10.1126/SCIENCE.AAN2556.

3. Jiang G, Giannone G, Critchley DR, Fukumoto E, Sheet MP. Two-piconewton slip bond between fibronectin and the cytoskeleton depends on talin. Nature. 2003;424:334–337. doi:10.1038/NATURE01805.

4. Roca-Cusachs P, Iskratsch T, Sheetz MP. Finding the weakest link-exploring integrin-mediated mechanical molecular pathways. Journal of Cell Science. 2012;125:3025–3038. doi:10.1242/JCS.095794/258415/AM/FINDING-THE-WEAKEST-LINK-EXPLORING-INTEGRIN.

5. Calderwood DA. Integrin activation. Journal of Cell Science. 2004;117:657–666. doi:10.1242/JCS.01014.

6. Li J, Springer TA. Energy landscape differences among integrins establish the framework for understanding activation. Journal of Cell Biology. 2018;217:397–412. doi:10.1083/JCB.201701169.

7. Miller AE, Hu P, Barker TH. Feeling Things Out: Bidirectional Signaling of the Cell–ECM Interface, Implications in the Mechanobiology of Cell Spreading, Migration, Proliferation, and Differentiation. Advanced Healthcare Materials. 2020;9:1901445. doi:10.1002/ADHM.201901445.

8. Ellis SJ, Lostchuck E, Goult BT, Bouaouina M, Fairchild MJ, López-Ceballos P, et al. The Talin Head Domain Reinforces Integrin-Mediated Adhesion by Promoting Adhesion Complex Stability and Clustering. PLoS Genetics. 2014;10. doi:10.1371/JOURNAL.PGEN.1004756.

9. Humphries JD, Wang P, Streuli C, Geiger B, Humphries MJ, Ballestrem C. Vinculin controls focal adhesion formation by direct interactions with talin and actin. The Journal of cell biology. 2007;179:1043–1057. doi:10.1083/JCB.200703036.

10. Roca-Cusachs P, Gauthier NC, del Rio A, Sheetz MP. Clustering of $\alpha 5\beta 1$ integrins determines adhesion strength whereas αv$\beta 3$ and talin enable mechanotransduction. Proceedings of the National Academy of Sciences of the United States of America. 2009;106:16245–16250. doi:10.1073/pnas.0902818106.

11. Choi CK, Vicente-Manzanares M, Zareno J, Whitmore LA, Mogilner A, Horwitz AR. Actin and alpha-actinin orchestrate the assembly and maturation of nascent adhesions in a myosin II motor-independent manner. Nature cell biology. 2008;10:1039–1050. doi:10.1038/ncb1763.

12. Changede R, Xu X, Margadant F, Sheetz MP. Nascent Integrin Adhesions Form on All Matrix Rigidities after Integrin Activation. Developmental cell. 2015;35:614–621. doi:10.1016/J.DEVCEL.2015.11.001.

13. Schvartzman M, Palma M, Sable J, Abramson J, Hu X, Sheetz MP, et al. Nanolithographic control of the spatial organization of cellular adhesion receptors at the single-molecule level. Nano Letters. 2011;11:1306–1312. doi:10.1021/NL104378F.

14. Yao M, Goult BT, Chen H, Cong P, Sheetz MP, Yan J. Mechanical activation of vinculin binding to talin locks talin in an unfolded conformation. Scientific Reports 2014 4:1. 2014;4:1–7. doi:10.1038/srep04610.

15. Yao M, Goult BT, Klapholz B, Hu X, Toseland CP, Guo Y, et al. The mechanical response of talin. Nature Communications 2016 7:1. 2016;7:1–11. doi:10.1038/ncomms11966.

16. Rio AD, Perez-Jimenez R, Liu R, Roca-Cusachs P, Fernandez JM, Sheetz MP. Stretching single talin rod molecules activates vinculin binding. Science. 2009;323:638–641. doi:10.1126/SCIENCE.1162912.

17. Gingras AR, Ziegler WH, Frank R, Barsukov IL, Roberts GCK, Critchley DR, et al. Mapping and Consensus Sequence Identification for Multiple Vinculin Binding Sites within the Talin Rod. Journal of Biological Chemistry. 2005;280:37217–37224. doi:10.1074/JBC.M508060200.

18. Stumpf BH, Ambriović-Ristov A, Radenovic A, Smith AS. Recent Advances and Prospects in the Research of Nascent Adhesions. Frontiers in Physiology. 2020;11:1562. doi:10.3389/FPHYS.2020.574371.

19. Cooper G. The Cell: A Molecular Approach. 2nd edition. Sinauer Associates; 2000.

20. Molloy JE, Burns JE, Kendrick-Jones B, Tregear RT, White DCS. Movement and force produced by a single myosin head. Nature 1995 378:6553. 1995;378:209–212. doi:10.1038/378209a0.

21. Kee YS, Robinson DN. Motor Proteins: Myosin Mechanosensors. Current Biology. 2008;18:R860–R862. doi:10.1016/j.cub.2008.07.071.

22. Chan CE, Odde DJ. Traction dynamics of filopodia on compliant substrates. Science (New York, NY). 2008;322:1687–1691. doi:10.1126/SCIENCE.1163595.

23. Franz CM, Müller DJ. Analyzing focal adhesion structure by atomic force microscopy. Journal of Cell Science. 2005;118:5315–5323. doi:10.1242/JCS.02653.

24. Cheng B, Lin M, Li Y, Huang G, Yang H, Genin GM, et al. An Integrated Stochastic Model of Matrix-Stiffness-Dependent Filopodial Dynamics. Biophysical Journal. 2016;111:2051. doi:10.1016/J.BPJ.2016.09.026.

25. Elosegui-Artola A, Bazellières E, Allen MD, Andreu I, Oria R, Sunyer R, et al. Rigidity sensing and adaptation through regulation of integrin types. Nature materials. 2014;13:631. doi:10.1038/NMAT3960.

26. Changede R, Sheetz M. Integrin and cadherin clusters: A robust way to organize adhesions for cell mechanics. BioEssays. 2017;39:1–12. doi:10.1002/BIES.201600123.

27. Vicente-Manzanares M, Zareno J, Whitmore L, Choi C, Horwitz AF. Regulation of protrusion, adhesion dynamics, and polarity by myosins IIA and IIB in migrating cells. Journal of Cell Biology. 2007;176:573–580. doi:10.1083/jcb.200612043.

28. Okeyo KO, Adachi T, Hojo M. Dynamic coupling between actin network flow and turnover revealed by flow mapping in the lamella of crawling fragments. Biochemical and Biophysical Research Communications. 2009;390:797–802. doi:10.1016/J.BBRC.2009.10.052.

29. Oser M, Condeelis J. The Cofilin Activity Cycle in Lamellipodia and Invadopodia. Journal of cellular biochemistry. 2009;108:1252. doi:10.1002/JCB.22372.

30. Webb DJ, Donais K, Whitmore LA, Thomas SM, Turner CE, Parsons JT, et al. FAK–Src signalling through paxillin, ERK and MLCK regulates adhesion disassembly. Nature Cell Biology 2004 6:2. 2004;6:154–161. doi:10.1038/ncb1094.

31. Elosegui-Artola A, Oria R, Chen Y, Kosmalska A, Pérez-González C, Castro N, et al. Mechanical regulation of a molecular clutch defines force transmission and transduction in response to matrix rigidity. Nature Cell Biology 2016 18:5. 2016;18:540–548. doi:10.1038/ncb3336.

32. Franco SJ, Rodgers MA, Perrin BJ, Han J, Bennin DA, Critchley DR, et al. Calpain-mediated proteolysis of talin regulates adhesion dynamics. Nature Cell Biology 2004 6:10. 2004;6:977–983. doi:10.1038/ncb1175.

33. Bhatt A, Kaverina I, Otey C, Huttenlocher A. Regulation of focal complex composition and disassembly by the calcium-dependent protease calpain. Journal of Cell Science. 2002;115:3415–3425. doi:10.1242/JCS.115.17.3415.

34. Bell GI. Models for the Specific Adhesion of Cells to Cells. Science. 1978;200:618–627. doi:10.1126/SCIENCE.347575.

35. Dembo M, Torney DC, Saxman K, Hammer D. The reaction-limited kinetics of membrane-to-surface adhesion and detachment. Proceedings of the Royal Society B: Biological Sciences. 1988;234:55–83. doi:10.1098/RSPB.1988.0038.

36. Choi YI, Duke-Cohan JS, Chen W, Liu B, Rossy J, Tabarin T, et al. Dynamic control of β1 integrin adhesion by the plexinD1-sema3E axis. Proceedings of the National Academy of Sciences of the United States of America. 2014;111:379–384. doi:10.1073/PNAS.1314209111.

37. Kong F, García AJ, Mould AP, Humphries MJ, Zhu C. Demonstration of catch bonds between an integrin and its ligand. Journal of Cell Biology. 2009;185:1275–1284. doi:10.1083/JCB.200810002.

38. Chen W, Lou J, Zhu C. Forcing Switch from Short- to Intermediate- and Long-lived States of the αA Domain Generates LFA-1/ICAM-1 Catch Bonds. Journal of Biological Chemistry. 2010;285:35967–35978. doi:10.1074/JBC.M110.155770.

39. Zhou DW, Lee TT, Weng S, Fu J, García AJ. Effects of substrate stiffness and actomyosin contractility on coupling between force transmission and vinculin–paxillin recruitment at single focal adhesions. Molecular Biology of the Cell. 2017;28:1901. doi:10.1091/MBC.E17-02-0116.

40. Novikova EA, Storm C. Contractile Fibers and Catch-Bond Clusters: a Biological Force Sensor? Biophysical Journal. 2013;105:1336–1345. doi:10.1016/J.BPJ.2013.07.039.

41. Owen LM, Bax NA, Weis WI, Dunn AR. The C-terminal actin-binding domain of talin forms an asymmetric catch bond with F-actin. Proceedings of the National Academy of Sciences of the United States of America. 2022;119. doi:10.1073/PNAS.2109329119.

42. Lavelin I, Wolfenson H, Patla I, Henis YI, Medalia O, Volberg T, et al. Differential Effect of Actomyosin Relaxation on the Dynamic Properties of Focal Adhesion Proteins. PLOS ONE. 2013;8:e73549. doi:10.1371/JOURNAL.PONE.0073549.

43. Bachir AI, Zareno J, Moissoglu K, Plow EF, Gratton E, Horwitz AR. Integrin-Associated Complexes Form Hierarchically with Variable Stoichiometry in Nascent Adhesions. Current Biology. 2014;24:1845–1853. doi:10.1016/J.CUB.2014.07.011.

44. Cheng B, Wan W, Huang G, Li Y, Genin GM, Mofrad MRK, et al. Nanoscale integrin cluster dynamics controls cellular mechanosensing via FAKY397 phosphorylation. Science Advances. 2020;6. doi:10.1126/SCIADV.AAX1909.
